# A Population of Radio-Resistant Macrophages in the Deep Myenteric Plexus Contributes to Postoperative Ileus *Via* Toll-Like Receptor 3 Signaling

**DOI:** 10.3389/fimmu.2020.581111

**Published:** 2021-01-13

**Authors:** Jana Enderes, Shilpashree Mallesh, Reiner Schneider, Kristof J. Hupa, Mariola Lysson, Bianca Schneiker, Kristian Händler, Balthasar Schlotmann, Patrick Günther, Joachim L. Schultze, Jörg C. Kalff, Sven Wehner

**Affiliations:** ^1^ Department of Surgery, Division of Immune Pathophysiology, University Hospital Bonn, Bonn, Germany; ^2^ PRECISE Platform for Single Cell Genomics and Epigenomics, German Center for Neurodegenerative Diseases (DZNE), University of Bonn, Bonn, Germany; ^3^ Genomics and Immunoregulation, Life & Medical Sciences (LIMES) Institute, University of Bonn, Bonn, Germany; ^4^ Systems Medicine, German Center for Neurodegenerative Diseases (DZNE), Bonn, Germany

**Keywords:** postoperative ileus, TLR3, TRIF, macrophages, innate immune response

## Abstract

Postoperative ileus (POI) is triggered by an innate immune response in the muscularis externa (ME) and is accompanied by bacterial translocation. Bacteria can trigger an innate immune response *via* toll-like receptor (TLR) activation, but the latter’s contribution to POI has been disproved for several TLRs, including TLR2 and TLR4. Herein we investigated the role of double-stranded RNA detection *via* TLR3 and TIR-domain-containing adapter-inducing interferon-β (TRIF) signaling pathway in POI. POI was induced by small bowel intestinal manipulation in wt, TRIF^-/-^, TLR3^-/-^, type I interferon receptor^-/-^ and interferon-β reporter mice, all on C57BL/6 background, and POI severity was quantified by gene expression analysis, gastrointestinal transit and leukocyte extravasation into the ME. TRIF/TLR3 deficiency reduced postoperative ME inflammation and prevented POI. With bone marrow transplantation, RNA-sequencing, flow cytometry and immunohistochemistry we revealed a distinct TLR3-expressing radio-resistant MHCII^hi^CX3CR1^-^ IBA-1^+^ resident macrophage population within the deep myenteric plexus. TLR3 deficiency in these cells, but not in MHCII^hi^CX3CR1^+^ macrophages, reduced cytokine expression in POI. While this might not be an exclusive macrophage-privileged pathway, the TLR3/TRIF axis contributes to proinflammatory cytokine production in MHCII^hi^CX3CR1^-^ IBA-1^+^ macrophages during POI. Deficiency in TLR3/TRIF protects mice from POI. These data suggest that TLR3 antagonism may prevent POI in humans.

## Introduction

Postoperative ileus (POI) is a gastrointestinal dysmotility and a common consequence of abdominal surgery. Patients suffer from nausea and vomiting, abdominal pain and oral food intolerance, which causes prolonged hospital stay and is associated with increased health care costs (1). The pathophysiology of POI is multifactorial and involves activation of resident immunocytes and cells of the enteric nervous system within the muscularis externa (ME) during surgery. Upon activation, these cells release proinflammatory cytokines and chemokines which further trigger and maintain ME inflammation and recruitment of blood-derived leukocytes (2). Resident ME macrophages (3, 4), dendritic cells (DC) (5, 6), and enteric glial cells (7) were shown to be important contributors in the initial phase of this inflammatory process. Macrophages highly express the CX3C chemokine receptor 1 (CX3CR1) (8) and show a prominent distribution within the intestinal musculature (9). Furthermore, they are self-maintaining and colonize distinct niches in the gut, and they are essential for intestinal homeostasis during which they can shape intestinal motility (8, 10).

Although crucial, the underlying molecular mechanisms of resident immunocyte activation during POI are still not fully understood. Possible triggers could be release of endogenous damage-associated molecular patterns (DAMPs) upon the surgical trauma as well as pathogen-associated molecular patterns that originate from luminal microbiota. Intestinal bacteria were shown to translocate into the ME and even disseminate to distant organs (11) due to a surgery-induced intestinal barrier dysfunction (12). The translocated bacteria exhibit multiple toll-like receptor (TLR) ligands and resident ME macrophages are known to express several TLRs (13). However, previous work from our group demonstrated that prominent TLRs, i.e., TLR2 and TLR4 (7), both detecting bacterial cell wall components as well as TLR9 were not involved in POI pathogenesis. While all the TLRs signal *via* the adapter molecule MyD88, TLR3 exclusively signals *via* the TIR-domain-containing adapter-inducing interferon-β (TRIF). In contrast to TLR2 and TLR4, TLR3 is an intracellular receptor, located on the endosomal compartment, and activated by double stranded RNA (dsRNA). As TLR3 can also be expressed on phagosomes upon phagosome-endosome fusion (14) and was already described to detect damaged cells upon sterile radio trauma to the intestine or the skin (15, 16) we hypothesized that TLR3 might play a role in POI pathogenesis.

In the present manuscript, we investigated the role of the TLR3-TRIF pathway in several transgenic and knockout mouse models, revealed the relevant molecular mechanisms of TLR3 activation upon surgery and identified a resident macrophage population colonizing a distinct anatomical niche in the ME as a responsible cell population for TLR3 signaling during POI.

## Methods

### Animals

Wildtype (wt) C57BL/6 mice were purchased from Janvier (Saint-Berthevin Cedex, France), TRIF^-/-^ mice (17), TLR3^-/-^ mice (18), IFNAR^-/-^ mice (19), IFN-βluc^+/-^ mice (20), CX3CR1^GFP/+^ mice (21), CX3CR1^GFP/+^ xTLR3^d/d^ mice (own breeding) and LysM^cre+^;ROSA26^LSL-eYFP^ (22), all on C57BL/6 background, were kept under specific pathogen-free conditions in the animal housing facility of the University of Bonn (Germany). Mice were acclimatized and cohoused with littermates in groups of up to five animals for one week after transportation from the vendor. Animal experiments were performed two to four times as indicated and with the indicated numbers of animals in female mice with a bodyweight between 20 to 25 g. Whenever possible we performed the interventions of different genotypes (e.g. intestinal manipulation of TLR^+/+^ and TLR^-/-^) on one day. Mice had free access to a standard diet chow and water ad libitum.

All animal experiments were performed according to the German Protection of Animals Act (TierSchG) and were approved by the governmental authority of North-Rhine Westfalia (LANUV).

### Generation of Bone Marrow Chimeric Mice

Bone marrow transfer was used to create chimeric mice with a TLR3 deficiency either in the radio-sensitive circulating cells (wt x TLR3^-/-^ chimera) or in the radio-resistant resident tissue cells (TLR3^-/-^ x wt). Bone marrow cells were collected from the femur and tibia of wt or TLR3^-/-^ donor mice. A total of 1.2 x 10^7^ cells were injected intravenously to 9-Gy irradiated host mice 6–7 h after irradiation. Four chimera groups were generated (wt x wt bone marrow, wt x TLR3^-/-^ bone marrow, TLR3^-/-^ x wt bone marrow and TLR3^-/-^ x TLR3^-/-^ bone marrow). To test if lethally irradiated MHCII^hi^CX3CR1^+^ and MHCII^hi^CX3CR1^-^ cells are replaced by circulating monocytes we either lead-shielded or did not shield the abdomen of CX3CR1^GFP/+^ mice before 9-Gy irradiation and reconstituted them 6–7 h later with 1.2 x 10^7^ bone marrow cells from the femur and tibia of LysM^cre+^;ROSA26^LSL-eYFP^ donor mice. Further analyses were performed 6–7 weeks after irradiation.

### Animal Model of Postoperative Ileus

POI was induced by a standardized intestinal manipulation (IM) procedure as described previously in detail^3^. In brief, Mice were anesthetized by inhalation of isoflurane (3%–5% isoflurane, 3–5 L/min flow), placed in a spine position and injected with Tramadol 10 mg/kg BW subcutaneously 5 min prior to surgery (as an additional analgesia postoperatively mice received Tramadol 1mg/ml with drinking water). A midline incision of the skin measuring 1 cm and opening of the abdominal muscles and the peritoneum along the linea alba was performed. The small intestine was carefully eventrated, placed on a sterile tissue and smoothly rolled between two moist cotton swabs twice antegradely from the duodenum to the terminal ileum. The intestine was neither transected nor sutured. After this mechanical manipulation procedure, the small intestine was then carefully placed back into the abdominal cavity. Peritoneum and skin were closed by two layers of continuous sutures. For controls, either sham-operated or non-operated mice were used, as indicated. Sham operation was performed accordingly to the before mentioned IM procedure including the eventration of the small bowel out of the belly but without the mechanical manipulation. The intestine was replaced into the belly after the same duration the manipulation took in the IM group.

### Gastrointestinal Transit

To analyze the effects of IM on gut motility gastrointestinal transit (GIT) was measured 24 h after IM as previously described (23). In brief, 22.5 h after manipulation mice were gavaged with 100 µl FITC-labeled dextran (70 kDa; 6.25 mg/ml/gavage). After a fasting period of 90 min organs were harvested 24 h after IM and the intestinal tract from stomach to colon was divided into 15 segments. Luminal contents were washed out to determine the absorbance of dextran. The data were expressed as the percentage of activity per intestinal segment. GIT was calculated as the geometric center (GC) of distribution of the fluorescence marker using the following formula: GC = Σ (% of total fluorescent signal per segment * segment number) ÷ 100.

### Histochemical and Immunohistochemical Analysis

Staining of MPO^+^ monocytes and neutrophils was performed 24 h after IM. Midjejunal, mucosal-free muscularis whole mounts were fixed in ethanol (Applichem, Darmstadt, Germany) for 10 min and stained with Hanker-Yates reagent as described previously (24). MPO^+^ cells were counted under a microscope (TE2000, Nikon, Düsseldorf, Germany) in 5 randomly chosen areas in each specimen at a 200x magnification and calculated as MPO^+^ cells/mm^2^.

For immunofluorescence stainings for CX3CR1, MHCII, βIII-tubulin and IBA-1 midjejunal, mucosal-free muscularis whole mounts of either untreated CX3CR1^GFP/+^ or irradiated/shielded or irradiated/non-shielded CX3CR1^GFP/+^ were fixed in 4% PFA for 20 min, permeabilized with 1% Triton X-1000 (Sigma), blocked with 5% donkey serum for 1 h and incubated with the appropriate antibodies at 4°C overnight. For antibodies used in this study see **Table 1**. All secondary antibodies were used at a 1:800 dilution and incubated for 1 h at room temperature. Nuclei were stained by HOECHST (Sigma), 3 µg/ml. Microscopic images were either taken by a TE2000 Nikon microscope at 200x magnification or on a Leica SP8 confocal microscope at 400x magnification.

**Table 1:** List of antibodies used in this study.

ProteinTarget	Dye/Dilution	Secondary antibody*	Use	Company
CD45	APC/1:200		FACS	eBioscience
CD45	Pacific blue/1:200		FACS	Biolegend
CD45	Cy5/1:200		FACS	eBioscience
MHCII	PE or PerCp-eF710/1:200		FACS	Biolegend
Ly6C	PE/Cy7/1:200		FACS	Biolegend
Ly6G	AlexaFluor647/1:300		FACS	eBioscience
CD103	PE/1:200		FACS	Biolegend
CD11c	PE/Cy7/1:200		FACS	Biolegend
CD11b	BV711/1:200		FACS	Biolegend
anti-CD16/32	1:50 (blocking)		FACS	Biolegend
Near-IR	APC/Cy7/1:200		FACS	Thermo Fisher
GFP	1:600	AlexaFluor488	IHC	Novus biologicals
MHCII	1:400	AlexaFluor647 or Cy3	IHC	BioLegend
βIII-Tubulin	1:600	Dylight405	IHC	BioLegend
IBA-1	1:200	AlexaFluor647	IHC	Abcam

*All secondary antibodies were raised in donkey and used at 1:800 dilution.

### Preparation of Single-Cell Suspension of the Muscularis Externa

Isolation of ME was achieved by sliding small bowel segments onto a glass rod and removing the outer muscularis circumferentially with moist cotton applicators. ME was then cut into fine pieces and digested with a 0.1% collagenase type II (Worthington Biochemical, Lakewood, NJ, USA) enzyme mixture, diluted in HBSS, containing 0.1 mg/ml DNase I (La Roche, Germany), 2.4 mg/ml Dispase II (La Roche, Germany), 1 mg/ml BSA (Applichem), and 0.7 mg/ml trypsin inhibitor (Applichem) for 40 min in a 37°C shaking water bath. Afterwards single cell suspension was obtained using a 70 µm filter mesh.

### Isolation of Resident Muscularis Externa Macrophages

For sorting of MHCII^hi^CX3CR1^+^ and MHCII^hi+^CX3CR1^-^ cell populations, ME of six or nine mice per group was pooled in order to collect enough cells for further analyses. Single cell suspension was prepared as described above. After incubation with FcR-block (anti-CD16/32, BioLegend) cells were stained for 30 min at 4°C with the appropriate antibodies. For antibodies used in this study see **Table 1**. Cell sorting was performed on FACSAriaIII (BD Bioscience) and different cell populations were captured in PBS with 10% FCS.

### Fluorescence Activated Cell Sorting

Fluorescence activated cell sorting (FACS) analysis was performed on isolated ME of the small bowel 3 and 24 h after intestinal manipulation and in untreated control animals as well as in lethally irradiated shielded and non-shielded CX3CR1^GFP/+^xLysM^cre+^;Rosa26^YFP^ chimera, respectively. Isolation of ME was achieved by sliding small bowel segments onto a glass rod, removing the outer muscularis circumferentially with moist cotton applicators and cutting the ME into fine pieces. ME was digested with a 0.1% collagenase type II (Worthington Biochemical, Lakewood, NJ, USA) enzyme mixture, diluted in HBSS, containing 0.1 mg/ml DNase I (La Roche, Germany), 2.4 mg/ml Dispase II (La Roche, Germany), 1 mg/ml BSA (Applichem), and 0.7 mg/ml trypsin inhibitor (Applichem) for 40 min in a 37°C shaking water bath. Afterwards single cell suspension was obtained using a 70 µm filter mesh. Cells were stained for 30 min at 4°C with the appropriate antibodies. For antibodies used in this study see **Table 1**. Flow cytometry analyses were performed on FACSCanto III (BD Biosciences) or with LSRFortessa™ (BD Biosciences) and Attune™ NxT (Invitrogen™), respectively, and data were analyzed with FlowJo software (Tree Star, Ashland, OR, USA).

### *In Vivo* Imaging System Analysis

Bioluminescence was measured in IFN-βluc^+/-^ and wt mice 3, 6, and 24 h after intestinal manipulation. Another set of IFN-βluc^+/-^ mice received an i.p. injection of 200 µg PolyI:C/mouse (Invivogen) or vehicle (sterile endotoxin-free physiological water, NaCl 0.9%). Bioluminescence was analyzed using an *In-Vivo* Imaging System (IVIS) 200 system (Caliper LifeSciences) 5 min after i.p. injection of luciferin (50 mM, Caliper Life Sciences) in PBS. Data analysis was performed with Living Image 2.50.1 software (Caliper LifeSciences).

### Enzyme-Linked Immunosorbent Assay

ME was harvested 15 min after surgery and snap frozen in liquid nitrogen. Release of ph-IκB was measured in ME RIPA lysates by Enzyme-Linked Immunosorbent Assay (ELISA) following the manufacturer’s instructions. The ELISA was purchased from Cell Signaling (PathScan, phIκBα (Ser32) Sandwich ELISA Kit #7355).

### Gene Expression Analysis

Total RNA was extracted from ME using the RNeasy Mini extraction kit (Qiagen, Hilden, Germany) followed by treatment with deoxyribonuclease I (Ambion, Austin, USA) and reverse transcribed to complementary DNA using the High Capacity cDNA Reverse Transcription Kit (Applied Biosystems, Darmstadt, Germany). Expression of messenger RNA (mRNA) was quantified in triplicate by a real-time reverse transcriptase polymerase chain reaction (RT-PCR). Gene expression data was normalized to the housekeeper gene GAPDH. For primer sequences, primer assays or TaqMan probes used see **Table 2**. Quantitative polymerase chain reaction was performed with SYBR Green PCR Master Mix (Applied Biosystems) or TaqMan Gene Expression Master Mix (Applied Biosystems).

**Table 2:** Primer sequences, primer assays and Taqman probes used for quantitative RT-PCR.

Gene name	Primer sequences (Metabion)	
	forward	reverse
EGR-1	5-GAGCGAACA	5-GGCCAGTAT
	ACCCTATGAGC-3	AGGTGATGGGA-3
ISG15	5-CCCCAGCAT	5-TGACTGTGA
	CTTCACCTTTA-3	GAGCAAGCAGC-3
USP18	5-GTGTCCGTG	5-CTGCAGAAA
	ATCTGGTCCTT-3	TACAACGTGCC-3
TLR3	5-GCCTGAATC	5-AGCCCAGAT
	ACAATCGCGCACC-3	TATGGGTGCAATCCCT-3
**Gene name**	**QuantiTect Primer Assay ID (Qiagen)**	
IL-1α	QT00113505	
IFN-β	QT00249662	
**Gene name**	**Taqman Probes Assay ID (Applied Biosystems)**	
IL-6	Mm00446190	
IL-1β	Mm00434228	
GAPDH	Mm99999915_g1	

### RNA-Seq Library Preparation, Sequencing

For RNA sequencing, 6–25x10^5^ cells were harvested for each sample and lysed in TRIzol (Invitrogen). Total RNA was extracted according to the manufacturer’s protocol. The precipitated RNA was re-suspended in RNase-free water and RNA quantity and quality (RIN) were assessed *via* the RNA analysis screen tape assay on a 2200 TapeStation system (Agilent Technologies). Total RNA was converted into double-stranded cDNA libraries as a template for high-throughput sequencing using the TruSeq RNA Sample Preparation Kit v2 (Illumina). cDNA libraries were quantified with the KAPA Library Quantification Kit (Kapa Biosystems). After cluster generation on a cBot (Illumina), a 75 bp single read, rapid run was performed on a HiSeq1500 (Illumina).

### RNA-Seq Data Analysis

After base calling and de-multiplexing using CASAVA version 1.8.2, data RNA-Seq libraries were subjected to initial quality control using FASTQC (http://www.bioinformatics.babraham.ac.uk/projects/fastqc, v0.11.7). Next, raw reads were pseudoaligned to the murine transcriptome (mm10, GENCODE general assembly release vM16) using Kallisto with default settings (25). Based on the pseudo alignment estimated by Kallisto, transcript levels were quantified and counts were imported into R using tximport (26). Transcript information was summarized on gene-level. We imported the resulting dataset and performed the downstream analysis using the DESeq2 pipeline [v1.2 (27)].

### Statistical Analysis

Statistical analysis was performed using Graph Pad Prism Version 8.4.3 for Windows software (GraphPad Software, San Diego, CA). Data were analyzed by two-way ANOVA with Tukey *post hoc* test, one-way ANOVA with Bonferroni *post hoc* test or a student’s t-test as indicated. The data are shown as the means ± SEM.

## Results

### TRIF Deficient Mice Are Protected From Postoperative Ileus

**Figure 1:**
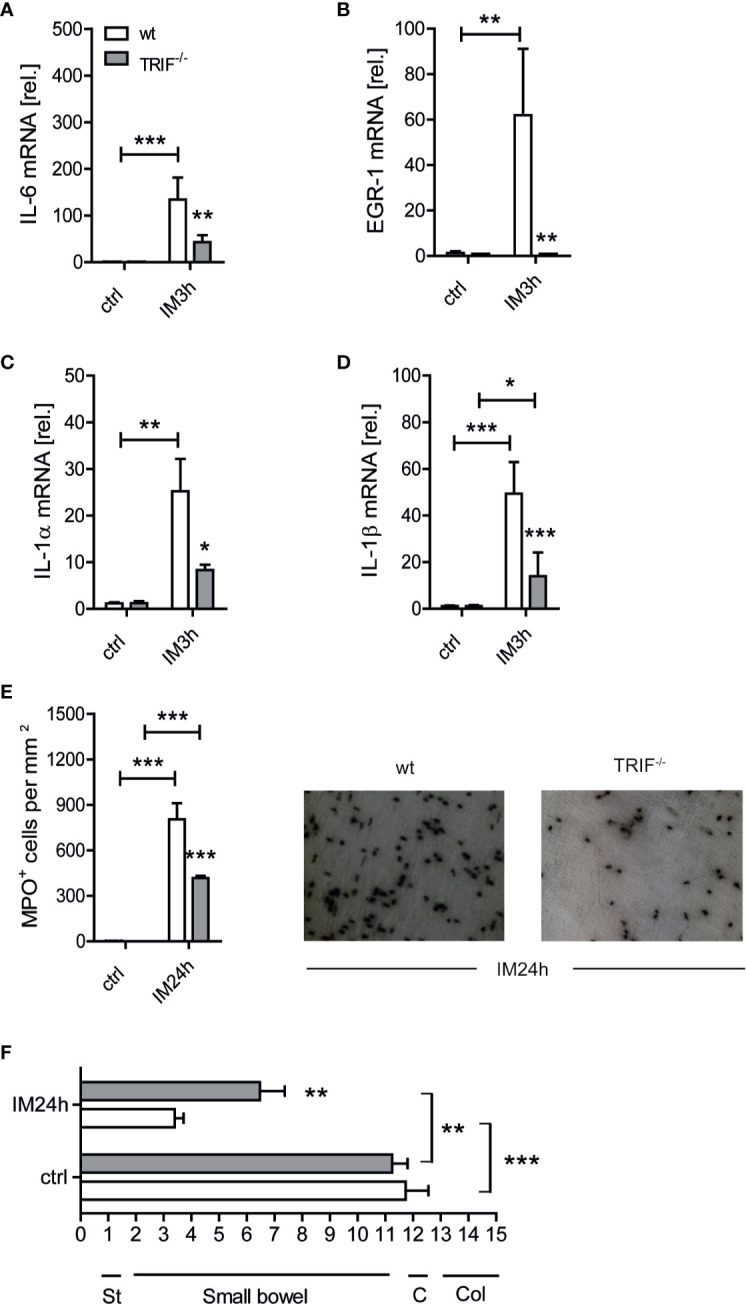
TIR-domain-containing adapter-inducing interferon-β (TRIF) deficient mice are protected from postoperative ileus (POI). Wt and TRIF^-/-^ mice underwent IM or were left untreated (ctrl). **(A–D)** Three hours after surgery (IM3h), IL-6, EGR-1, IL-1α, and IL-1β gene expression levels were analyzed within the muscularis externa (ME) and displayed as fold changes after normalization to ctrl. **(E)** MPO^+^ leukocyte numbers were identified by Hanker-Yates reaction in mucosal-free muscularis whole mounts 24 h after surgery (IM24h). **(F)** Gastrointestinal (GI) transit time was determined by distribution of a fluorescent meal along the GI tract within a defined time period. Data were calculated as geometric center of the fluorescence marker distribution. Geometric center (GC) values reciprocally correlate to the motility. St, stomach; C, cecum; Col, colon. Experiments were performed two to four times reaching an overall size of n = 3–10 animals per group. Means + SEM were generated from the overall numbers of each group. Samples were analyzed by two-way analysis of variance with Tukey *post hoc* test and the results are displayed as means ± SEM. *p ≤ 0.05, **p ≤ 0.01, ***p ≤ 0.001 versus the corresponding wt group or between indicated groups.

In a first set of experiments, we investigated the role of TRIF in a standardized POI model. Wt and TRIF^-/-^ mice were subjected to a surgical IM procedure and small bowel ME specimens were analyzed for gene expression of different cytokines or MPO^+^ leukocyte infiltration 3 or 24 h after IM, respectively. After 3 h, IL-6 (p ≤ 0.001), EGR-1 (p ≤ 0.01), IL-1α (p ≤ 0.01), and IL-1β (p ≤ 0.001) were increased (**Figures 1A–D**) in intestinally manipulated wt animals compared to naive controls (ctrl) and TRIF^-/-^ mice. The latter showed a reduced expression of IL-6 (p ≤ 0.01), EGR-1 (p ≤ 0.01), IL-1α (p ≤ 0.05) and IL-1β (p ≤ 0.001). After 24 h, MPO^+^ cell counts were increased (p ≤ 0.001) in wt and in TRIF^-/-^ mice, but TRIF deficiency resulted in reduced infiltration (p ≤ 0.001) compared to the wt IM group (**Figure 1E**). Correspondingly, the gastrointestinal transit (GIT) of wt mice was decreased 24 h after IM compared to ctrl (p ≤ 0.001) while intestinally manipulated TRIF^-/-^ mice had an accelerated GIT (p ≤ 0.01) (**Figure 1F**). These data indicate that TRIF plays a crucial role in POI.

### TLR3 Deficient Mice Are Protected From Postoperative Ileus

**Figure 2:**
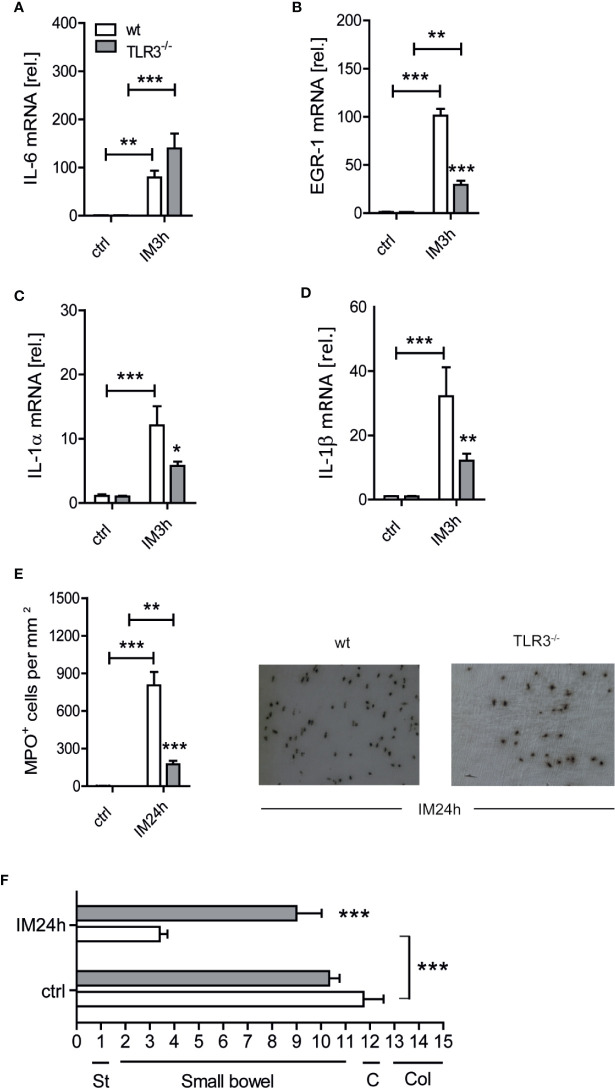
Toll-like receptor 3 (TLR3) deficient mice are protected from postoperative ileus (POI). Wt and TLR3^-/-^ mice underwent IM or were left untreated (ctrl). **(A–D)** Three hours after surgery (IM3h), IL-6, EGR-1, IL-1α and IL-1β gene expression levels were analyzed within the muscularis externa (ME) and displayed as fold changes after normalization to ctrl. **(E)** MPO^+^ leukocyte numbers were identified by Hanker-Yates reaction in mucosal-free muscularis whole mounts 24 h after surgery (IM24h). **(F)** Gastrointestinal (GI) transit time was determined by distribution of a fluorescent meal along the GI tract within a defined time period. Data were calculated as geometric center of the fluorescence marker distribution. Geometric center (GC) values reciprocally correlate to the motility. St, stomach; C, cecum; Col, colon. Experiments were performed two to four times reaching an overall size of n = 3–13 animals per group. Means + SEM were generated from the overall numbers of each group. Samples were analyzed by two-way analysis of variance with Tukey *post hoc* test and the results are displayed as means ± SEM. *p ≤ 0.05, **p ≤ 0.01, ***p ≤ 0.001 versus the corresponding wt group or between indicated groups.

TRIF is recruited after TLR3 or TLR4 ligation. As we previously discovered that TLR4 deficient mice were not protected from POI and that postoperative cytokine expression and leukocyte infiltration were also unaffected (7) in TLR4 deficient mice we focused our further investigations on TLR3 signaling. Again, in the intestinally manipulated wt animals IL-6 (p ≤ 0.01), EGR-1 (p ≤ 0.001), IL-1α (p ≤ 0.001), and IL-1β (p ≤ 0.001) gene expression was increased after 3 h (**Figures 2A–D**). Intestinally manipulated TLR3^-/-^ mice showed a diminished production of ph-IκB (p ≤ 0.05; **Supplementary Figure S1**), which resulted in a reduced upregulation of EGR-1 (p ≤ 0.001), IL-1α (p ≤ 0.05) and IL-1β (p ≤ 0.01) while IL-6 was slightly increased (p=0.059). Infiltrating MPO^+^ cells were reduced in TLR3^-/-^ mice (p ≤ 0.001) compared to the wt IM group (**Figure 2E**) and the GIT was accelerated (p ≤ 0.001) (**Figure 2F**). Together, these data demonstrate the involvement of a TLR3-TRIF signaling axis in postoperative ME inflammation in POI.

### A Type I IFN Response Does Not Contribute to Postoperative Ileus

**Figure 3:**
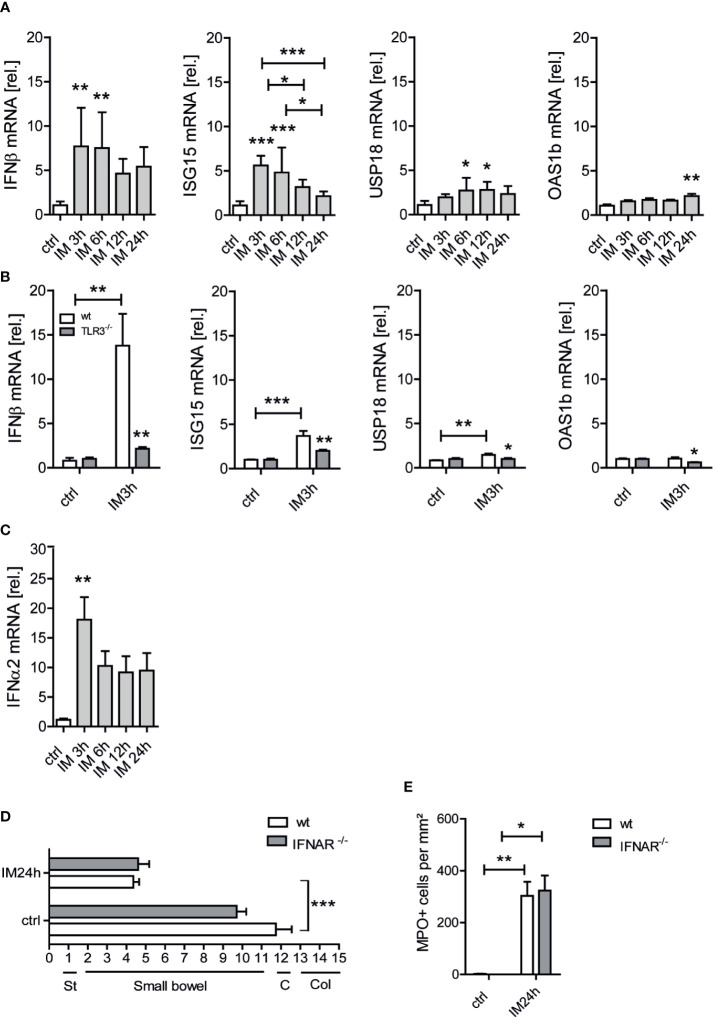
A type-I IFN response does not contribute to postoperative ileus (POI). Wt and TLR3^-/-^ mice underwent IM or were left untreated (ctrl). **(A, B)** IFN-β and interferon stimulated genes (ISGs) ISG15, USP18, and OAS1b as well as **(C)** IFNα2 gene expression were analyzed at the indicated time points within the ME and displayed as fold changes after normalization to ctrl. n = 7 for all groups. Samples were analyzed by one-way analysis of variance or two-way analysis of variance with Bonferroni or Tukey *post hoc* test, respectively, and the results are displayed as means ± SEM. *p ≤ 0.05, **p ≤ 0.01, ***p ≤ 0.001 versus indicated groups. (D–E) Wt and IFNAR^-/-^ mice underwent intestinal manipulation (IM) or were left untreated (ctrl). **(D)** Gastrointestinal (GI) transit time was determined by distribution of a fluorescent meal along the GI tract within a defined time period. Data were calculated as geometric center of the fluorescence marker distribution. Geometric center (GC) values reciprocally correlate to the motility. St, stomach; C, cecum; Col, colon. **(E)** MPO^+^ leukocyte numbers were identified by Hanker-Yates reaction in mucosal-free muscularis whole mounts 24 h after surgery (IM24h). St, stomach; C, cecum; Col, colon. Experiments were performed two to three times reaching an overall size of n = 7 animals per group. Means + SEM were generated from the overall numbers of each group. Samples were analyzed by two-way analysis of variance with Tukey *post hoc* test and the results are displayed as means ± SEM. *p ≤ 0.05, **p ≤ 0.01, ***p ≤ 0.001 versus the corresponding wt group or between indicated groups.

TLR3 has been well elucidated to be involved in both, the production of proinflammatory cytokines and the induction of type I IFNs, with the latter particularly occurring after viral or bacterial infections. While our data show that the cytokine response is diminished in TLR3 deficient mice, it was unclear if a type I IFN response might also contribute to POI pathogenesis. Therefore, we first determined if wt mice develop a type I IFN signature upon IM. IFN-β as well as the IFN-stimulated genes (ISGs) ISG15 and USP18 were upregulated at different time points up to 12 h after IM while OAS1b was only slightly upregulated after 24 h (**Figure 3A**). In TLR3^-/-^ mice, this IM-mediated induction of IFN-β (p ≤ 0.01), ISG15 (p ≤ 0.01) and USP18 (p ≤ 0.05) was decreased (**Figure 3B**). IFN-α2, another type I IFN, was also upregulated within the immediate phase (**Figure 3C**). As the upregulation of ISGs was less pronounced compared to the type I interferons itself, we aimed to confirm type I IFN production on the protein level. IFN-βluc^+/-^ mice were subjected either to IM, to an i.p. injection of polyI:C or were left untreated. While polyI:C treated mice demonstrated an IFN-β-promotor driven luciferase response at 3, 6, and 24 h after injection (**Supplementary Figures S2A,B**), intestinally manipulated mice as well as naive mice did not show any luciferase activity *in vivo*, demonstrating that IFN-β protein is not produced during POI. In order not to overlook any effects of other type I IFNs we additionally subjected IFNAR^-/-^ mice, which are unable to respond to all type I IFNs, to IM. Again, we did not observe any difference in GIT reduction between IFNAR^-/-^ and wt mice, neither between the naive nor between the IM groups (**Figure 3D**), and numbers of infiltrating MPO^+^ leukocytes were comparably increased in IFNAR^-/-^ and wt mice (**Figure 3E**). Furthermore, flow cytometry analysis of CD45^+^ (p=0.15), Ly6G^-^Ly6C^+^ (p=0.07), Ly6G^+^Ly6C^+^ (p=0.34) and F4/80^+^Ly6G^-^ (p=0.77) cells in the postoperative ME did not reveal any differences between IFNAR^-/-^ and wt mice (**Supplementary Figure S3**). Collectively, our data demonstrate that type I IFNs do not contribute to POI’s pathophysiology, indicating that the protective effect of TRIF or TLR3 deficiency is mediated by the reduced pro-inflammatory cytokine response.

### TLR3 Signals *Via* Resident Radio-Resistant Tissue Cells

**Figure 4:**
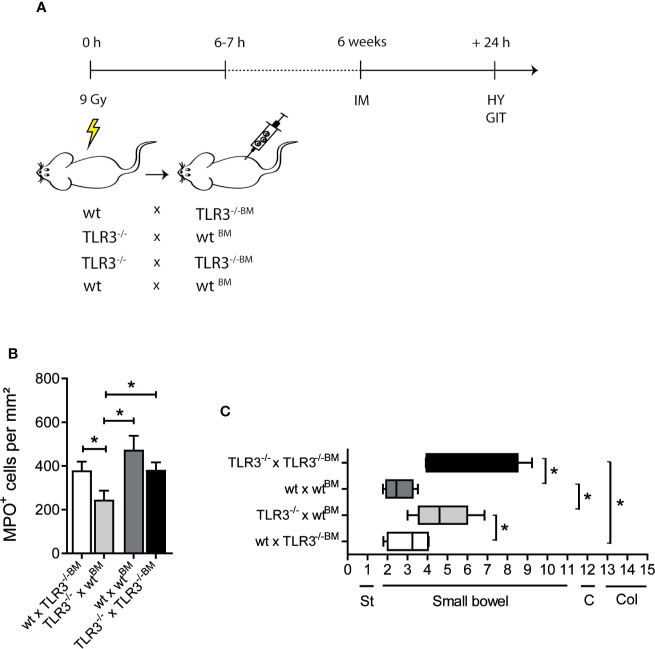
Toll-like receptor 3 (TLR3) signals *via* resident radio-resistant tissue cells. **(A)** Lethally irradiated host mice were recovered 6–7 h after radiation with a total of 1.2 x 10^7^ bone marrow (BM) cells. Four chimeric groups were generated (wt x TLR3^-/-BM^, TLR3^-/-^ x wt^BM^, TLR3^-/-^ x TLR3^-/-BM^, and wt x wt^BM^). Six weeks later, all four groups underwent intestinal manipulation (IM). **(B)** MPO^+^ leukocyte numbers were identified by Hanker-Yates (HY) reaction in mucosal-free muscularis whole mounts 24 h after surgery (IM24h). TLR3^-/-^ that received wt bone marrow showed lower leukocyte infiltration compared to wt mice that received TLR3^-/-^ bone marrow. **(C)** Gastrointestinal (GI) transit time was determined by distribution of a fluorescent meal along the GI tract within a defined time period. Data were calculated as geometric center of the fluorescence marker distribution. Geometric center (GC) values reciprocally correlate to the motility. St, stomach; C, cecum; Col, colon. Experiments were performed two times reaching an overall size of n = 6 animals per group. Means + SEM were generated from the overall numbers of each group. Samples were analyzed by two-way analysis of variance with Tukey *post hoc* test and the results are displayed as means ± SEM. *p ≤ 0.05 versus indicated groups.

Next, we aimed to further characterize the relevant TLR3 signaling cell type in POI and performed a bone marrow (BM) transplantation experiment in which either wt mice or TLR3^-/-^ underwent lethal irradiation followed by an injection of healthy bone marrow from TLR3^-/-^ (wt x TLR3^-/-BM^) or wt mice (TLR3^-/-^ x wt^BM^), respectively. Irradiation control groups were wt mice that received wt bone marrow (wt x wt^BM^) and TLR3^-/-^ mice that received TLR3^-/-^ bone marrow (TLR3^-/-^ x TLR3^-/-BM^) (**Figure 4A**). If TLR3 signaling involves local resident cells, that should be predominantly radio-resistant cells, one would expect a reduced postoperative leukocyte infiltration in TLR3^-/-^ x wt^BM^. In turn, involvement of bone marrow-derived cells, which are known to be radio-sensitive and give rise to blood leukocytes that infiltrate the postoperative ME, would rather become obvious by a reduced cell infiltration in wt x TLR3^-/-BM^ mice. Indeed, TLR3^-/-^ x wt^BM^ showed decreased numbers of infiltrating MPO^+^ cells (p ≤ 0.05) compared to wt x TLR3^-/-BM^ 24 h after IM (**Figure 4B**). In accordance, the GIT of TLR3^-/-^ x wt^BM^ was improved (p ≤ 0.05) compared to wt x TLR3^-/-BM^ 24 h after IM (**Figure 4C**). Furthermore, MPO^+^ cells in TLR3^-/-^ x wt^BM^ compared to wt x wt^BM^ were also significantly decreased (p ≤ 0.05) and accordingly, these mice showed an improved GIT (p ≤ 0.05). Notably, this improvement was comparable to the accelerated GIT of intestinally manipulated TLR3^-/-^ x TLR3^-/-BM^ mice compared to the wt x wt^BM^ group. Taken together, these data indicate that TLR3 signaling rather occurs in radio-resistant (mostly likely tissue resident) cells than in radio-sensitive (most likely infiltrating blood-derived) cells during POI.

### Radio-Resistant MHCII^hi^CX3CR1^-^ Cells Express the Macrophage Marker IBA-1 and Reside Within a Special Anatomical Niche

**Figure 5:**
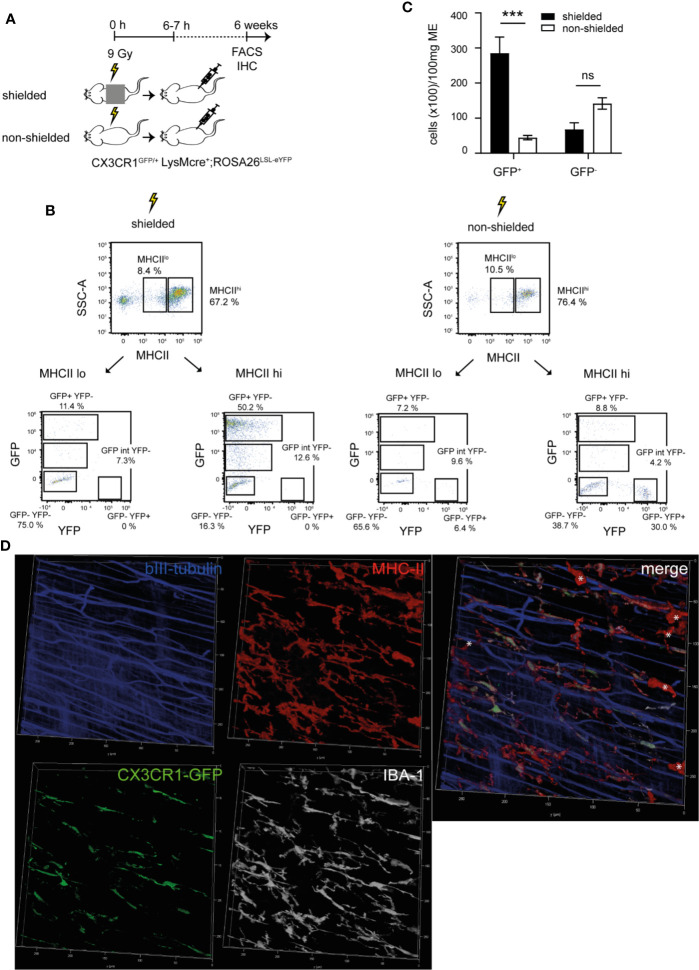
Radio-resistant MHCII^hi^CX3CR1^-^ cells express the macrophage marker IBA-1 and reside within a special anatomical niche. **(A–C)** Lethally irradiated shielded and non-shielded CX3CR1^GFP/+^ were recovered 6–7 h after radiation with a total of 1.2 x 10^7^ bone marrow (BM) cells of LysM^cre+^;ROSA26^LSL-eYFP^ donor mice. Six weeks later, fluorescence activated cell sorting (FACS) analysis and whole mount immunohistochemistry (IHC) was performed. **(A)** Scheme of the experimental setup. **(B)** Representative FACS plots of the expression of GFP (CX3CR1)/YFP (LysM) on living CD45^+^ MHCII^hi^/MHCII^lo^ gated cells isolated from the ME with Invitrogen™ Attune™ NxT in shielded and non-shielded CX3CR1^GFP/+^ x LysM^cre+^;ROSA26^LSL-eYFP^ and **(C)** quantification of total CD45^+^MHCII^hi^GFP^+^ and CD45^+^MHCII^hi^GFP^-^ cells. **(D)** Representative confocal image stack of CX3CR1^GFP/+^ stained for GFP (green), MHCII (red), βIII-tubulin (blue) and IBA-1 (gray) revealing the existence of a MHCII^hi^IBA-1^+^CX3CR1^-^ cell population (*) located in a different layer than the MHCII^hi^IBA-1^+^CX3CR1^+^ cells. Experiments were performed three times reaching an overall size of n = 3 animals per group. Means + SEM were generated from the overall numbers of each group. Samples were analyzed by student’s t-test and the results are displayed as means ± SEM. ***p ≤ 0.001. For images on the localization, please refer to **Supplementary Figure S7**.

Although TLR3 can be expressed by a variety of cell types, it is mainly expressed on leukocytes. In the intestinal ME, a dense network of CX3CR1^+^ macrophages is known to express TLRs and their contribution to POI is common knowledge (28, 29). Early work demonstrated that depletion and inactivation of these cells ameliorates POI Considering our observation that TLR3 and TRIF demonstrated reduced immune mediator transcripts already in the early phase of POI, we expected that these resident macrophages, rather than the later infiltrating blood-derived monocytes, might be the relevant cell population of TLR3 signaling. However, in this case and in line with the bone marrow chimera experiments these cells should be - at least to a significant part - radio-resistant. To test this hypothesis we lethally irradiated belly-shielded and non-shielded CX3CR1^GFP/+^ chimeras and reconstituted them with BM of LysM^cre+^;ROSA26^LSL-eYFP^ mice (**Figure 5A**). Replacement of CX3CR1^GFP/+^ cells by bone marrow derived LysM-expressing YFP^+^ leukocytes would indicate if the resident CX3CR1^+^ macrophages are radio-resistant or to which extent they might become replaced by bone-marrow derived progenitors. Six weeks after BM transplantation recipients were analyzed for the presence of GFP^+^ and YFP^+^ cells in the ME (for gating strategies see **Supplementary Figure S4**). After irradiation and BM transplantation we detected an overall decrease in MHCII^hi^GFP^+^ cells in non-shielded versus shielded mice (shielded: 50.2%, non-shielded: 8.8%) and an influx of YFP^+^ cells in irradiated non-shielded animals (shielded: 0%; non-shielded: 30.0%) (**Figure 5B**). While MHCII^hi^GFP/CX3CR1^+^ cells were reduced upon irradiation, no reduction among MHCII^hi^GFP/CX3CR1^-^ cells was observed (**Figure 5C**). The overall number of CD45^+^ cells was not altered (**Supplementary Figure S5**) and additionally immunohistochemical quantification of ileal whole mount specimen in shielded and non-shielded irradiated CX3CR1^GFP/+^ mice upon LysM^cre+^;ROSA26^LSL-eYFP^ BM transplantation confirmed that also the overall number of MHCII^+^ cells was not altered. Notably, the MHCII staining included both, GFP^+^ and YFP^+^ cells (**Supplementary Figure S6**), indicating that the loss in MHCII^hi^CX3CR1^+^ cells, detected by flow cytometry, might be compensated by MHCII^hi^ leukocytes that were or became CX3CR1^-^. As all MHCII^+^ cells appear in their typical stellate/bipolar morphology, we assume that the YFP^+^ BM derived cells indeed replaced CX3CR1^+^ cells after irradiation.

We next aimed to precisely locate the MHCII^hi^CX3CR1^-^ population within the ME of naive CX3CR1^GFP/+^ mice. Confocal immunohistochemistry with antibodies directed against GFP, expressed under the control of the CX3CR1 promotor and against MHCII indeed identified three different MHCII^+^ immunocyte populations (**Figure 5D**). Two CX3CR1^+^ cell populations in close proximity to beta-III tubulin^+^ enteric neurons of which one presented in a bipolar and the other one in a stellate-shaped morphology. These cells have already been characterized as resident macrophages of the serosal and myenteric plexus, respectively. The third MHCII^+^CX3CR1^-^ population was also stellate-shaped but located underneath both CX3CR1^+^ populations. Notably, the CX3CR1^-^ population was also located in close proximity to beta-III tubulin^+^ enteric neurons. A 3D projection of the confocal images indeed confirmed the distinct anatomical niche distant to the CX3CR1^+^ cell layers. This layer separation is shown in **Supplementary Figure S7** and we conclude that MCHII^+^CX3CR1^-^ cells lie in the deep myenteric plexus of the circular muscle layer. Importantly, a simultaneous IBA-1 immunostaining revealed that all MHCII^+^ cells, including the CX3CR1^-^ population, were highly IBA-1 positive (**Figure 5D** **;** **Supplementary Figure S7**), indicating that these cells are also resident macrophages. Additional flow cytometry analysis also demonstrated that the vast amount of the CX3CR1^-^ was CD11b positive. Only a very small amount of these cells expressed CD11c and the prototypical DC marker CD103 (**Supplementary Figure S8**). Taken together, these data demonstrate that MHCII^hi^CX3CR1^+^ resident ME macrophages are radio-sensitive while a MHCII^hi^CX3CR1^-^ cell population presented with a radio-resistant phenotype. The latter are located in a distinct anatomical niche, the deep myenteric plexus, and express the common resident macrophage marker IBA-1.

### MHCII^hi^CX3CR1^-^ Cells Have a Unique Toll-Like Receptor Gene Expression Pattern

**Figure 6:**
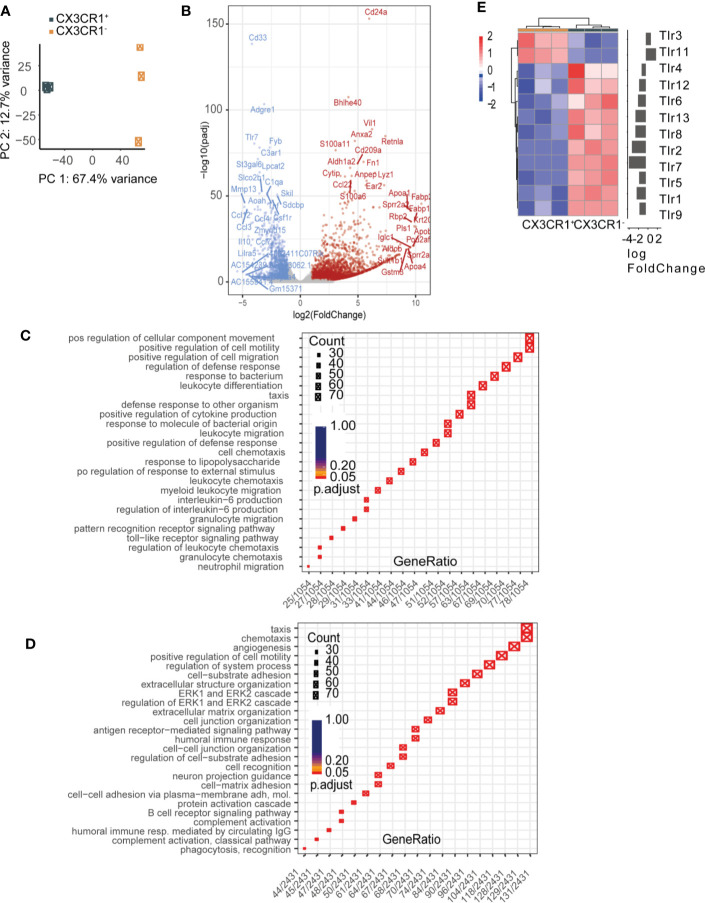
Radio-resistant MHCII^hi^CX3CR1^-^ cells express toll-like receptor 3 (TLR3). MHCII^hi^CX3CR1^+^ and MHCII^hi^CX3CR1^-^ cell populations were flow cytometry sorted from the muscularis externa (ME) of naive CX3CR1^GFP/+^ mice and underwent bulk RNA-seq. **(A)** Principal component analysis (PCA) and hierarchical clustering of samples confirming a segregation of CX3CR1^+^ and CX3CR1^-^ populations according to their gene expression profiles. **(B)** Volcano plot showing significantly (adj. p-value < 0.05) differentially expressed genes as upregulated (FoldChange> 1, red) or downregulated (FoldChange < 1, blue) genes. Gray genes do not pass differentially expressed thresholds. Top 15 most highly significant genes are labeled for up and downregulated genes. **(C, D)** Visual representation of GO terms associated with enriched genes versus *gene ratio* showing genes downregulated (C) and upregulated D in CX3CR1^-^ cells compared to CX3CR1^+^ cells. **(E)** Heatmap of TLR genes showing a unique expression pattern of TLR, with TLR3 and TLR11 being predominantly expressed on CX3CR1^-^ cells. n = 3 (ME of six mice per group was pooled).

As our bone-marrow chimera experiment revealed a particular role for TLR3 deficiency in radio-resistant cells, we hypothesized that the CX3CR1^-^ cells might be responsible for the TLR3-triggered cytokine response in POI. Following this hypothesis, we sorted MHCII^hi^CX3CR1^-^ and MHCII^hi^CX3CR1^+^ cells from the ME of naive CX3CR1^GFP/+^ mice and compared their transcriptome by RNA sequencing (bulk RNA-seq). Principal component analysis (PCA) and hierarchical clustering of samples confirm a segregation of CX3CR1^+^ and CX3CR1^-^ populations according to their gene expression profiles. While CX3CR1^+^ cells clustered very closely, we observed some segregation in the CX3CR1^-^ cell population (**Figure 6A**) which indicates a certain heterogeneity. While the list of most differentially expressed genes also contained DC marker genes, we analyzed the MHCII^hi^CX3CR1^-^ cells by flow cytometry for CD103, CD11b and CD11c expression, however, only a very small amount of cells expressed CD11c and CD103 while most cells were only positive for CD11b, confirming their myeloid origin (**Supplementary Figure S8**). These data indicate a very low and probably negligible contamination of the CX3CR1^-^ macrophage population by a distinct DC fraction.

A total of 4,265 genes (|logFC|≥ log(2); ≤0.05 adjusted p-value) were differentially expressed between MHCII^hi^CX3CR1^+^ and MHCII^hi^CX3CR1^-^ cells (**Figure 6B**, **Supplementary Figure S9A**). A list of the top 25 up- and downregulated genes is shown in **Supplementary Figure S9B**. Notably, although CX3CR1^-^ cells expressed lower CSF1R levels than CX3CR1^+^ cells they showed higher levels of Ear2, Fn1, and Lys1, which are characteristic markers of the monocyte-macrophage lineage. Next, we performed functional annotation of differentially expressed genes by gene set enrichment analysis. This shows that genes upregulated in CX3CR1^-^ cells are associated with gene ontology terms including chemotaxis, ERK-signaling and cell-cell adhesion (**Figure 6D**). Genes that are downregulated in CX3CR1^-^ compared to CX3CR1^+^ show significant enrichment for gene ontology terms including cell migration, defense response and IL-6 production (**Figure 6C**). Genes listed under the GO term positive regulation of cell migration where also differentially expressed. Interestingly, we found a unique TLR expression pattern, with TLR3 and TLR11 being predominantly expressed on CX3CR1^-^ cells while all the other TLRs were mainly expressed on CX3CR1^+^ cells (**Figure 6E**).

### TLR3 Signaling in MHCII^hi^CX3CR1^-^ Cells Drives Cytokine Expression During POI

**Figure 7:**
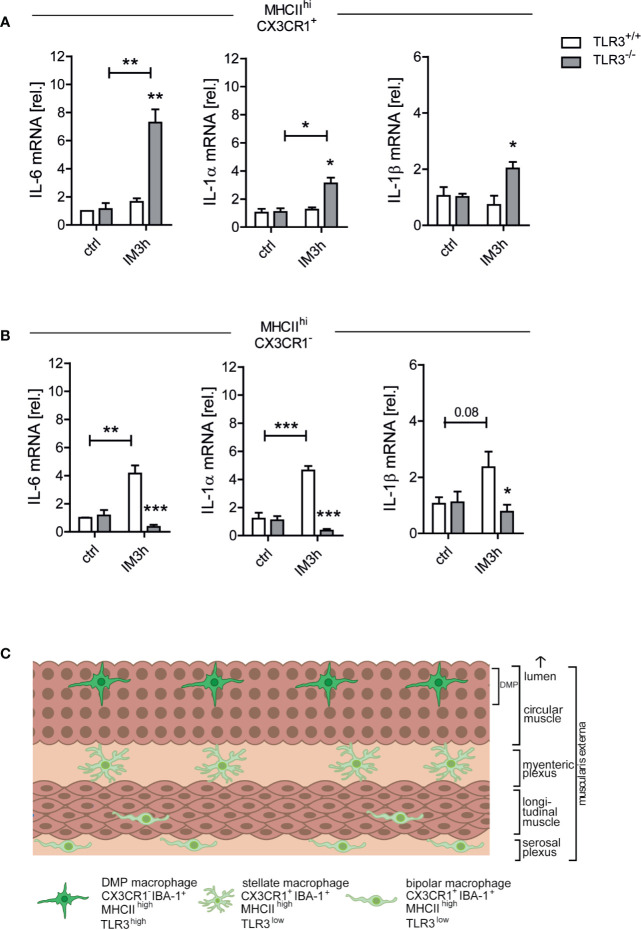
Toll-like receptor 3 (TLR3) signaling in MHCII^hi^CX3CR1^-^ cells drives cytokine expression during POI. Wt and TLR3^-/-^ mice underwent IM or were left untreated (ctrl). Three h after surgery (IM3h), gene expression levels of IL-6, IL-1β, and IL-1α were analyzed on **(A)** MHCII^hi^CX3CR1^-^ and **(B)** MHCII^hi^CX3CR1^+^ cell populations that had been sorted beforehand from the ME samples *via* fluorescence activated cell sorting (FACS) and displayed as fold changes after normalization to ctrl. n = 3 (each replicate was pooled from six mice). Samples were analyzed by two-way analysis of variance with Tukey *post hoc* test and student’s t-test, respectively, and the results are displayed as means ± SEM. Experiments were performed three times reaching an overall size of n = 3 animals per group. Means + SEM were generated from the overall numbers of each group. *p ≤ 0.05, **p ≤ 0.01, ***p ≤ 0.001 versus the corresponding TLR3^+/+^ group or indicated groups. **(C)** Schematic drawing of the intestinal muscularis externa (ME) illustrating the location of bipolar or stellate MHCII^hi^CX3CR1^+^ macrophages and MHCII^hi^CX3CR1^-^ macrophages. The latter reside within a distinct anatomical niche, the deep myenteric plexus (DMP), and express higher TLR3 levels while the MHCII^hi^CX3CR1^+^ cells are located within the myenteric and serosal plexus and show low TLR3 expression. Intestinal manipulation (IM) triggers a proinflammatory cytokine expression during the onset phase of postoperative ileus (POI) mainly in MHCII^hi^CX3CR1^-^ macrophages and this mechanism depends on TLR3 signaling.

In a final experiment, we aimed to investigate whether the early cytokine increase during POI indeed depends on TLR3 signaling in the MHCII^hi^CX3CR1^-^ cells. Unfortunately, neither TLR3-floxed mice nor cell-specific markers for the CX3CR1^-^ population, which might be used to drive Cre expression to achieve a cell-type specific TLR3 depletion, did exist. Therefore we decided to sort TLR-deficient CX3CR1^+^ and CX3CR1^-^ cells from CX3CR1^GFP/+^ xTLR3^-/-^ mice 3 h after IM and from naive controls to analyze gene expression of proinflammatory cytokines known to be involved in POI. Control cells were sorted from CX3CR1^GFP/+^ xTLR3^+/+^ littermates. Notably, as we were strongly limited in the amount of viable cells after sorting we were not able to perform more comprehensive transcriptome analyses, particularly not in the MHCII^hi^CX3CR1^-^ population. None of the tested genes were upregulated after IM in the CX3CR1^+^ population in TLR3 competent mice (**Figure 7A**), while all of them were induced in CX3CR1^-^ cells (IL-6, p ≤ 0.01; IL-1α, p ≤ 0.001; IL-1β, p=0.08) (**Figure 7B**). Unexpectedly, we observed an upregulation in IL-6 (p ≤ 0.01), IL-1α (p ≤ 0.05) and IL-1β (p ≤ 0.05) in the TLR3^-/-^ MHCII^hi^CX3CR1^+^ population after IM, which was not detected in MHCII^+^CX3CR1^-^ cells. In contrast, in the latter TLR3 deficiency resulted in a strong reduction in postoperative IL-6 (p ≤ 0.001), IL-1α (p ≤ 0.001) and IL-1β (p ≤ 0.05) compared to TLR3^+/+^ MHCII^hi^CX3CR1^-^ cells and levels did not differ from controls. These data indicate a specific role of TLR3 signaling in CX3CR1^-^ cells in the early cytokine expression during POI. Although TLR3 is much lower expressed in CX3CR1^+^ cells (log2FC = 1.2178578; non-log2FC = 2.326011; p-value = 9.919200e-07), it seems to fulfill a different, rather inhibitory role, in the postoperative cytokine production.

In conclusion, our results show that IM triggers a TLR3 dependent proinflammatory cytokine expression during the onset phase of POI in MHCII^hi^CX3CR1^-^ cells. Deficiency in TLR3 or downstream acting factor TRIF protects mice from POI. The MHCII^hi^CX3CR1^-^ cells express IBA-1, a common resident macrophage marker, and reside in a specialized anatomical niche, the deep myenteric plexus (for a schematic illustration see **Figure 7C**).

## Discussion

In the present study, we demonstrate that the TRIF-TLR3 pathway plays a prominent role in POI by triggering the postoperative cytokine response within the ME. Additionally, we also uncovered a subset of radio-resistant CD45^+^MHCII^hi^CX3CR1^-^ macrophages to be responsible for this TLR3-mediated cytokine production.

Our results underline that POI is driven by a cytokine response which has been previously shown to play a crucial role in POI development (30, 31), including IL-1α, IL-1β (7, 32), IL-6 (33), and Egr-1 (34). While IL-1 and Egr-1 were less induced in the postoperative ME of both, TRIF^-/-^ and TLR3^-/-^ mice, IL-6 was only reduced in TRIF^-/-^ but not in TLR3^-/-^ mice after IM. Therefore, the production of IL-6 mRNA seems to be at least in some cells independent of TLR3 signaling. Indeed, we observed a reduction of IL-6 mRNA also in TLR4^-/-^ (7) indicating a certain involvement of TLR4 in IL-6 production with TLR4 also acting as an upstream receptor in the TRIF pathway. Notably, TLR4^-/-^ mice did not show any improvement of POI. Although we can only speculate why TLR4^-/-^ mice are not protected from POI, a lack of TLR4 ligands seems to be unlikely due to the fact that bacteria can translocate into the ME upon IM (11). A counterbalance of the MyD88 pathway toward the TRIF pathway or intrinsic inhibitory effects of TRIF as described for TF5 - a TRIF-derived peptide - that inhibits TLR4 in a mouse model of LPS-induced inflammation (35) might be a possible explanation for this observation.

Alongside the cytokine release, we also observed an upregulation of a transcriptional type I IFN signature that was reduced in TLR3^-/-^ mice. In a series of experiments, we proved that this transcriptional type I IFN signature finally did not result in IFN-β production and does not play a role in POI. The discrepancy between the transcriptional type I response and the lack of protein production might simply be explained by DNA contaminations in the mRNA preparations, as type I IFN genes lack introns (20) and RT-PCR cannot distinguish between genomic DNA or cDNA templates. Furthermore, type I IFNs and ISGs show protective but also harmful effects (36) and thus type I IFN response is tightly controlled at signaling, transcriptional and translational levels. To this end, IFN response control mechanisms such as the expression of specific microRNA are able to negatively regulate IFN-induced signaling through a Stat3 pathway (37) and USP18 and ISG15 were described to supress type I IFN-mediated responses by negatively regulating JAK/STAT (38). Both pathways are activated during POI (23). Although we solely analyzed protein expression of IFN-β but not from ISGs or other type I interferons, we interpret the discrepancy in the elevated transcriptional type I IFN signature and the missing protein production as a protective mechanism to prevent an excessive immune response. The absence of any alteration in the postoperative immune cell infiltration and functional response during POI in IFNAR^-/-^ mice, which lack any type I interferon response, supports this conclusion.

TLR expression along intestinal epithelium is well described, and recently it was shown that the presence of TLRs differs along the gastrointestinal tract (GI tract) (39). However, less is known about the cellular site of TLR3 expression in the ME and so far - in the GI tract—it has mostly been studied by immunohistochemistry and any knowledge about the role of TLR3 signaling and possible contributions to immune responses during POI are missing. Our data indicate that radio-resistant rather than radio-sensitive cells contribute to TLR3 signaling during POI. Alongside multiple immunocytes and epithelial cells of the mucosa, enteric neurons and glial cells express TLR3 (40) and as structural cells these cells might be radio-resistant. Notably, especially enteric glial cells - as non-classical immunocytes - are also involved in POI’s pathogenesis and the underlying mechanism involves the release of IL-6 and MCP-1 *via* an MyD88 and IL1R1-mediated pathway (7). In contrast, resident muscularis macrophages are well known to induce and to orchestrate the inflammatory process in the postoperative ME (4) and were already described to express TLRs such as TLR2 and TLR4 (41). Furthermore, they are self-maintaining and only a minority undergoes replacement by bone marrow derived cells in naive mice (10). Therefore, we speculated that these resident macrophages might be responsible for TLR3/TRIF-mediated effects in POI and investigated whether these cells are radio-resistant. By use of CX3CR1^GFP/+^ mice, which express GFP in resident macrophages (8), we confirmed the previously described regular distribution of resident ME macrophages in the small bowel ME (9). Surprisingly, by co-labeling of MHCII, another highly expressed marker of murine and human ME macrophages (42), we not only identified MHCII^+^CX3CR1^+^ macrophages but also a population of MHCII^+^CX3CR1^-^ cells. Like the CX3CR1^+^ macrophages, these cells also expressed IBA-1, a well-known marker for resident microglia and macrophages, which also stains resident macrophages in the ME (43). 3D reconstructions of confocal images revealed that these cells lay in close proximity to beta-III tubulin^+^ enteric nerves in a layer underneath the CX3CR1^+^ cells (closer to the gut lumen), which are mainly found in the myenteric and serosal plexus (9). From these findings, we conclude that the MHCII^hi^CX3CR1^-^ cells are a distinct population of resident macrophages localized in the deep myenteric plexus. Notably, a lack of CX3CR1^+^ macrophages within the deep myenteric plexus has also just recently been observed by others (10). Additional evidence about a distinct macrophage subpopulation in the deep myenteric plexus comes from the Mikkelsen group, which already identified a specialized IBA-1^+^ CD169^-^ population in the deep myenteric plexus (44). Interestingly, our RNA-seq data also revealed strongly downregulated CD169 levels in the CX3CR1^-^ population.

With regard to the radio-sensitivity of both resident macrophage populations we found a reduction of CX3CR1^+^ cells in the ME with a simultaneous increase of YFP^+^ bone-marrow derived cells in irradiated CX3CR1^GFP/+^ mice. As not all MHCII^hi^CX3CR1^+^ cells were replaced by YFP^+^ cells, this population might be sensible in terms of radio-sensitivity or may even consist of different, so far non-distinguishable subtypes of macrophages. An alteration of the self-maintenance of CX3CR1^+^ ME macrophages and bone-marrow replacement was also recently shown in another bone-marrow transplantation study wherein the CX3CR1^+^ resident cells were depleted by a diphtheria-toxin driven genetic approach (10). Although this study did not investigate if CX3CR1^+^ cells of the ME are radio-resistant, it shows that these cells in general can be replenished by bone-marrow cells after genetic depletion. This is in line with our findings which identified LysM-YFP^+^ bone-marrow derived cells as the source replenishing CX3CR1^+^ resident cells after irradiation. Notably, six weeks after irradiation, all MHC^+^ cells displayed microscopically a prototypical bipolar or stellate cell morphology, indicating that the infiltrating LysM-YFP^+^ cells gave rise to fully differentiated resident macrophages. In contrast and more importantly, the MHCII^hi^CX3CR1^-^ macrophages were not affected by the irradiation and remained present in the ME. This indicates that these cells are radio-resistant in nature what qualifies these cells to be involved in TLR3/TRIF signaling in POI. The reason for the difference in radio-sensitivity of MCHII^hi^CX3CR1^-^ and MCHII^hi^CX3CR1^+^ immunocytes remains unclear. Maybe the different anatomical locations and morphologies (9, 45) play a role in the fate of these cells upon irradiation.

Our RNA-seq analyses provide further evidence on a distinct macrophage phenotype of the MHCII^hi^CX3CR1^-^ population. Gene expression patterns significantly differed and importantly CX3CR1^-^ cells showed a unique TLR gene expression signature with increased TLR3 and TLR11 expression levels, compared to MHCII^hi^CX3CR1^+^ macrophages, which show increased levels of all other TLRs. While our RNA-seq analysis also detected higher transcript levels of the common DC markers CD103 and Flt3 in MHCII^hi^CX3CR1^-^ cells compared to their CX3CR1^+^ neighbors, we analyzed both populations by flow cytometry for presence of CD103, CD11b and CD11c. Notably, a previous study identified also a small population of resident CD11b^+^CD103^+^ DCs within the intestinal muscularis (46). However, only a very small percentage of MHCII^hi^CX3CR1^-^ cells expressed the DC markers while the vast majority only stained positive for CD11b, confirming their myeloid origin.

In the last experimental setting, we demonstrated that MHCII^hi^CX3CR1^-^ cells upregulate inflammatory cytokines upon IM, while the same population of TLR3 deficient mice failed to upregulate these cytokines. Although the cytokines tested in our study have been shown to play significant roles in POI (7, 32, 33), they are not exclusively responsible for POI development. It should be noted that the absence of IL-6 expression in these cells was not observed in full ME specimens of intestinally manipulated TLR3^-/-^ compared to control mice (**Figure 2**). However, these data are not conflicting, as we have previously shown that enteric glial cells can also release IL-6 during POI (7). Unexpectedly, the CX3CR1^+^ cell population of TLR3 competent mice did not show an elevated inflammatory cytokine expression. However, under TLR3 deficiency, they expressed IL-6, IL-1α, and IL-1β upon IM. This indicates that under TLR3-deficiency, other pathways become activated and take part in the postoperative cytokine production. Furthermore, our data reflect only one time point and therefore we cannot completely exclude a role of TLR3 competent CX3CR1^+^ cells in cytokine production at later time points in POI. Additionally, contribution of other radio-resistant cell types, known to express TLR3, e.g., enteric glial cells or neurons cannot be excluded. Conditional TLR3 knockout mice could provide clarity on the significance of TLR3 deficiency in any of these cells but these mice have so far not been available to us.

Finally, the question of the TLR3 ligand during POI still remains open. Since we were unable to detect dsRNA with a specific antibody by immunohistochemistry (clone J2, data not shown), we can only speculate on its source, which might be either viral, in the form of replication intermediaries from many enteral viruses (47), or cellular double-stranded secondary mRNA (e.g., hair pins) that may be released from necrotic cells. This cellular dsRNA was shown to induce a TLR3 dependent inflammatory cytokine production *via* the NF-κB activation *in vitro* (48). A possible involvement of this cellular dsRNA in POI pathogenesis is supported by the observed partially reduced ph-IκB levels in TLR3-deficient mice, which shows that ph-IκB production is in part TLR3-dependent. Also, a recent study from our group showed a role of DAMPs released in the early phase of POI (32). In another trauma model, upon irradiation, dying intestinal cells were also shown to release dsRNA that led to TLR3-dependent crypt cell death and gastrointestinal syndrome. Blockage of TLR3/dsRNA led to a reduction of cell death and an improvement with regard to gastrointestinal syndrome (15). Not only in the gastrointestinal tract but also outside of it in the skin dsRNA from damaged cells was shown to induce hair follicle regeneration *via* TLR3 (16). In contrast, a viral origin of dsRNA is supported by detection of microbial translocation upon intestinal surgery in mice (11, 12) and in humans (49). With more than >10^12^ particles, bacteriophages represent the majority of human gut viruses (50). As they infect and reside within bacteria and can be released from them in response to stress signals (51), it appears not unlikely that they translocate within bacteria into the surgically manipulated ME.

In conclusion, this study reveals TLR3/TRIF signaling as a unique TLR pathway in the pathogenesis of POI, triggering inflammatory cytokine production in resident MHCII^hi^CX3CR1^-^IBA-1^+^ macrophages, which mainly colonize the deep myenteric plexus. Although further studies are needed to identify the source of dsRNA, this pathway is a promising new target for POI prevention.

## Data Availability Statement

The datasets presented in this study can be found in online repositories. The name of the repository and accession number can be found here: https://www.ncbi.nlm.nih.gov/, GSE132147.

## Ethics Statement

The animal study was reviewed and approved by the governmental authority of North-Rhine Westfalia (LANUV).

## Author Contributions

JE, KJH, SM, RS, ML, BS, and SW performed the research. JE, KJH, SM, RS, KH, BSch, PG, JCK, and SW analyzed the data, and JE and SW prepared the manuscript. SW designed the research. JLS critically read the manuscript. All authors contributed to the article and approved the submitted version.

## Funding

This study was funded by the Deutsche Forschungsgemeinschaft (DFG, German Research Foundation) under Germany’s Excellence Strategy – EXC 2151 – 390873048 and by a personnel grant of the German research council (DFG) to SW (WE4204).

## Conflict of Interest

SW and JCK receive royalties from Wolter Kluwer for contribution to the postoperative ileus section of the UpToDate library.

The remaining authors declare that the research was conducted in the absence of any commercial or financial relationships that could be construed as a potential conflict of interest.

## Abbreviations

CX3CR1, CX3C chemokine receptor 1; DAMPS, damage-associated molecular patterns, dsRNA, double stranded RNA; DC, dendritic cells; GIT, gastrointestinal transit; GI tract, gastrointestinal tract; GC, geometric center; IM, intestinal manipulation; ISGs, IFN-stimulated genes; ME, muscularis externa; MPO, myeloperoxidase; POI, postoperative ileus; TLR, toll-like receptor; TRIF, TIR-domain-containing adapter-inducing interferon-β.
