# Supplementary material for: A Population of Radio-Resistant Macrophages in the Deep Myenteric Plexus Contributes to Postoperative Ileus Via Toll-Like Receptor 3 Signaling
Source: Front Immunol. 2021 Jan 13;11:581111. doi: 10.3389/fimmu.2020.581111 (PMC7838642; doi:10.3389/fimmu.2020.581111)
Supplement: Supplementary file 1 [file DataSheet_1.pdf]

Supplementary Material

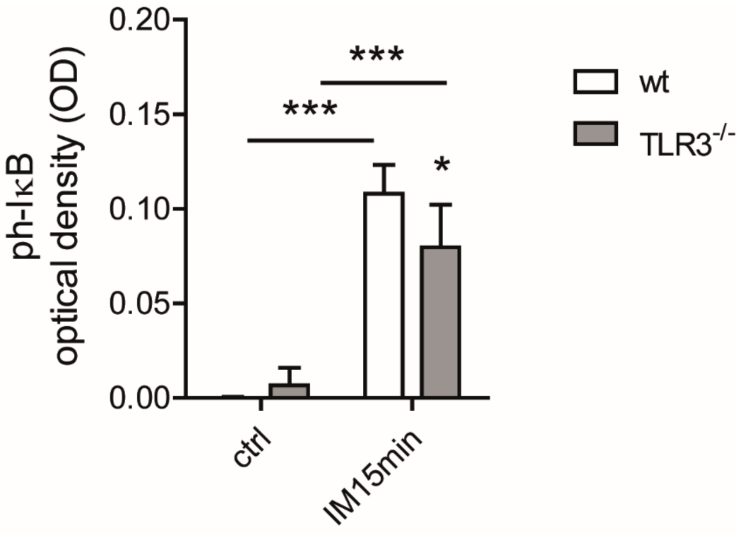

Supplementary Figure S1. Wt and $TLR3^{-/-}$ underwent IM. ME was isolated 15 min after surgery and snap frozen in liquid nitrogen. Release of ph-IκB was measured with ME RIPA lysates by ELISA following the manufacturer's instructions. n = 6 for all groups. Samples were analyzed by 2-way analysis of variance with Tukey post-hoc test and the results are displayed as means ± SEM. *p≤0.05, ***p≤0.001 versus indicated groups.

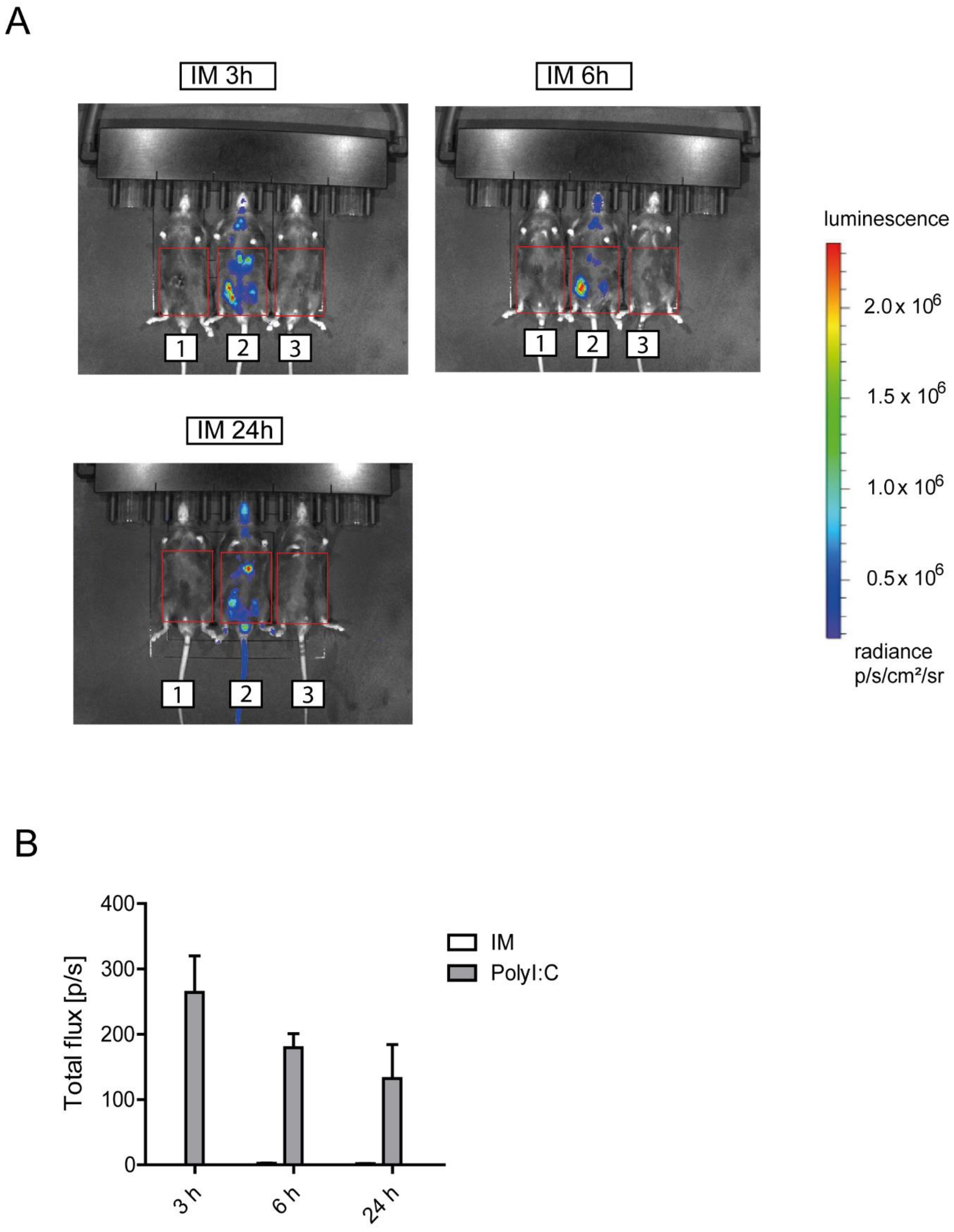

Supplementary Figure S2. (**A**) Bioluminescence was measured in IFN-βluc$^{+/-}$ (mouse 1 & 2) and wildtype (mouse 3) mice 3, 6 or 24 hrs after IM (mouse 1 and 3) or after i.p. injection of 200µg PolyI:C (mouse 2) using an IVIS 200 system (Caliper LifeSciences) 5 min after i.p. injection of luciferin (50 mM, Caliper Life Sciences) in PBS. (**B**) Data from dataset A were analyzed using the LivingImage software. n= 3 animals per group, 1 experiment.

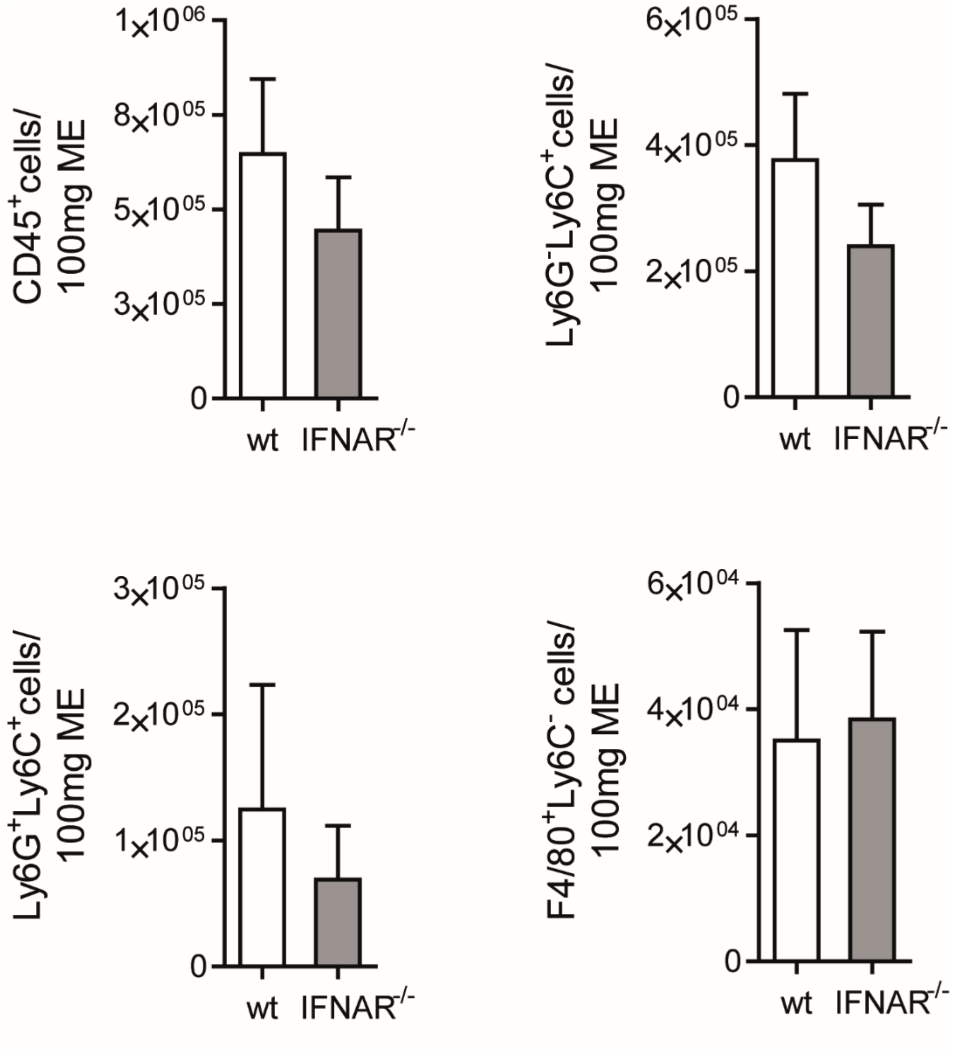

Supplementary Figure S3. Wt and IFNAR$^{-/-}$ mice underwent IM. In ME 24h after surgery absolute cell counts of CD45^{+} leukocytes, Ly6G-Ly6C^{+} monocytes, Ly6G^{+}Ly6C^{+} neutrophils and F4/80^{+}Ly6C^{-} resident macrophages were analyzed. n = 7 for all groups. Samples were analyzed by student's t-test, and the results are displayed as means ± SEM. *p≤0.05, **p≤0.01, ***p≤0.001 versus indicated groups.

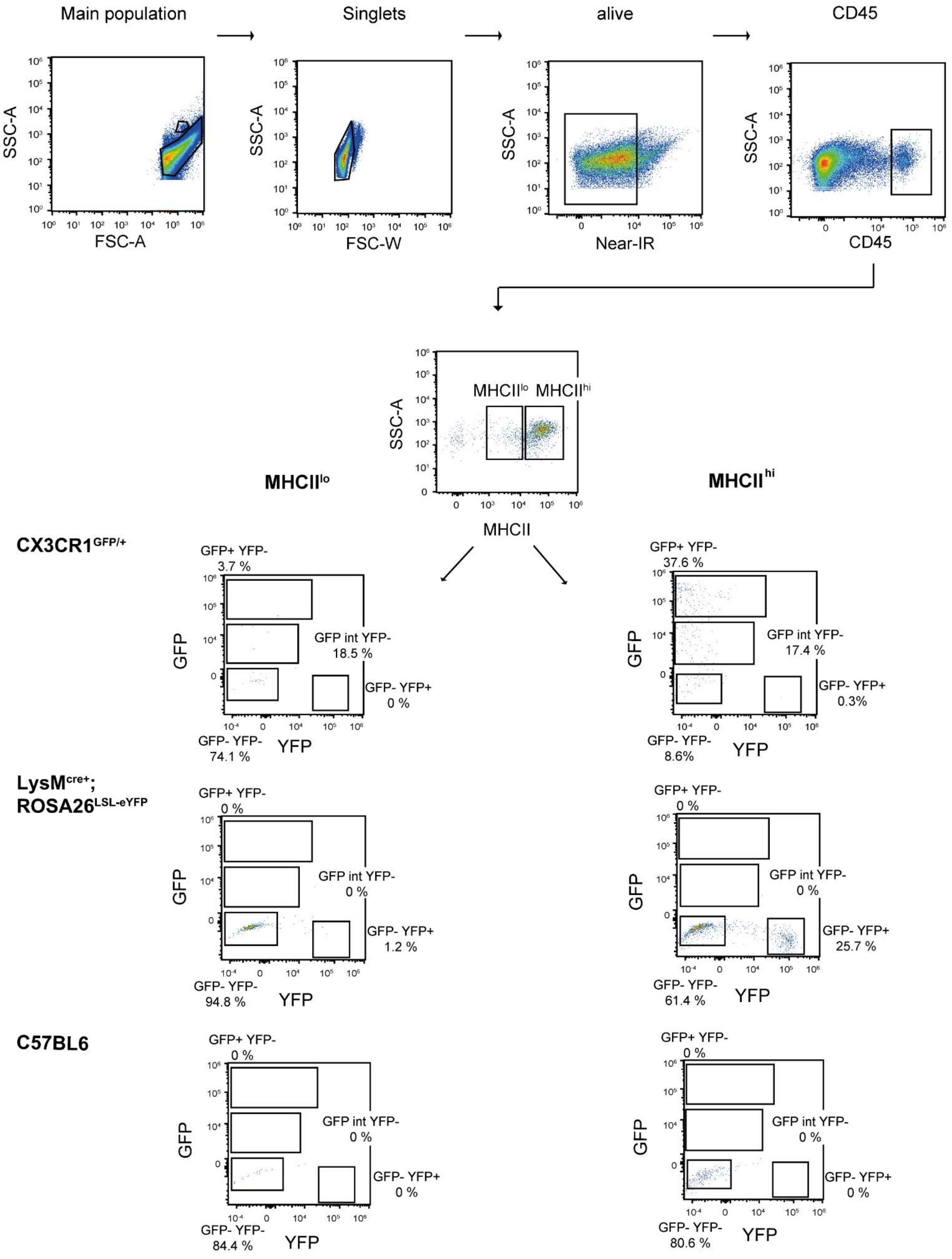
Main population
Singlets
alive
CD45
SSC-A
FSC-A
FSC-W
Near-IR
MHCII^{lo}
MHCII^{hi}
MHCII
CX3CR1$^{GFP/+}$
LysM^{cre+};
ROSA26$^{LSL\text{-}eYFP}$
C57BL6
GFP
YFP
GFP+ YFP-
3.7 %
GFP int YFP-
18.5 %
GFP- YFP+
0 %
GFP- YFP-
74.1 %
GFP+ YFP-
37.6 %
GFP int YFP-
17.4 %
GFP- YFP+
0.3%
GFP- YFP-
8.6%
GFP+ YFP-
0 %
GFP int YFP-
0 %
GFP- YFP+
1.2 %
GFP- YFP-
94.8 %
GFP+ YFP-
0 %
GFP int YFP-
0 %
GFP- YFP+
25.7 %
GFP- YFP-
61.4 %
GFP+ YFP-
0 %
GFP int YFP-
0 %
GFP- YFP+
0 %
GFP- YFP-
84.4 %
GFP+ YFP-
0 %
GFP int YFP-
0 %
GFP- YFP+
0 %
GFP- YFP-
80.6 %

Supplementary Figure S4. Representative gating strategy on MHCII expression levels of $CD45^+$ cells from the ME of $CX3CR1^{GFP/+}$ mice, $LysM^{cre+}$;$ROSA26^{LSL\text{-}eYFP}$ and C57BL6 mice. Note that the majority of the $CX3CR1^+$ macrophages is present in the $MHCII^{hi}$ population. Therefore, we followed this $MHCII^{hi}$ population in our further analysis.

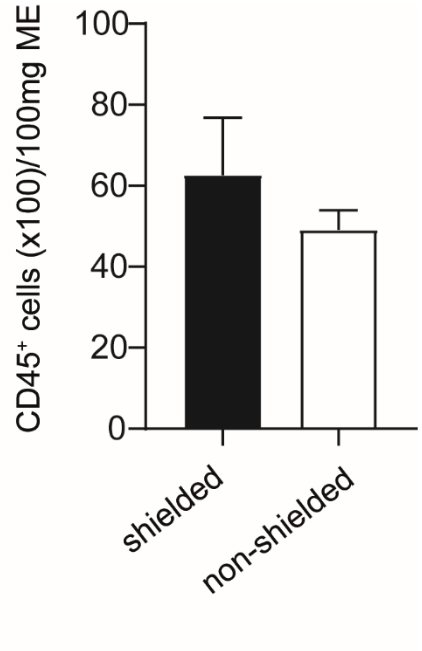

Supplementary Figure S5. Lethally irradiated shielded and non-shielded $CX3CR1^{GFP/+}$ were recovered 6-7h after radiation with a total of 1.2 x 107 bone marrow (BM) cells of $LysM^{cre+}$;$ROSA26^{LSL\text{-}eYFP}$ donor mice. 6 weeks later, FACS analysis and quantification of total $CD45^+$ cells in shielded and non-shielded mice was performed. n=3. Samples were analyzed by student's t-test and the results are displayed as means ± SEM.

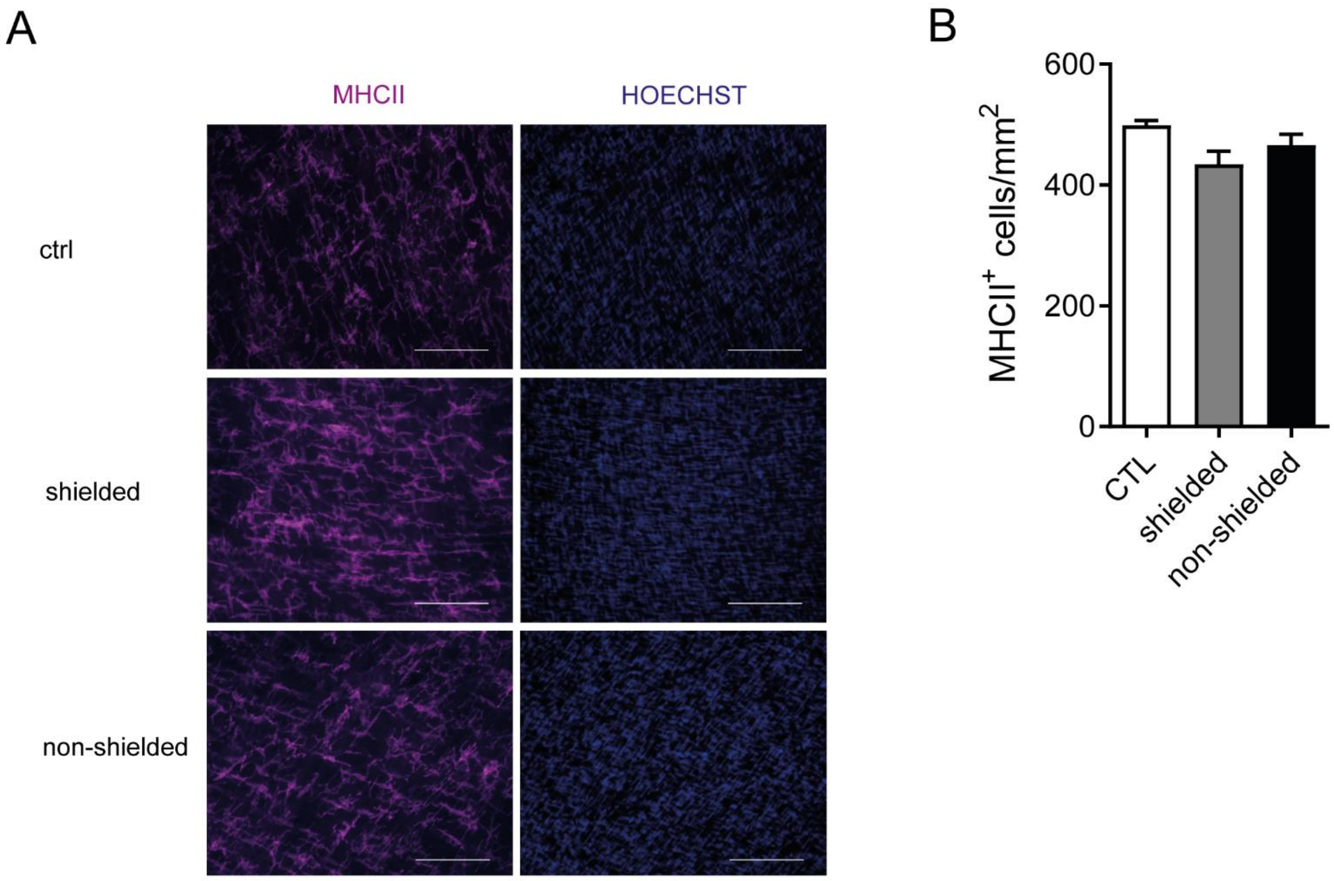

Supplementary Figure S6. Immunohistochemical analysis of ileal whole mount specimen in naive controls (ctrl), lethally irradiated shielded and non-shielded CX3CR1$^{GFP/+}$ mice that were recovered with a total of 1.2 x 10^{7} bone marrow cells of LysM^{cre+};ROSA26$^{LSL\text{-}eYFP}$ donor mice. 6 weeks later immunhistochemistry was performed. (**A**) Representative whole mounts were stained with MHCII (purple) and Hoechst (blue), scale bars 100µm and (**B**) quantification of total MHCII^{+} cells was performed. n=3 mice per group. Samples were analyzed by student's t-test and the results are displayed as means ± SEM.

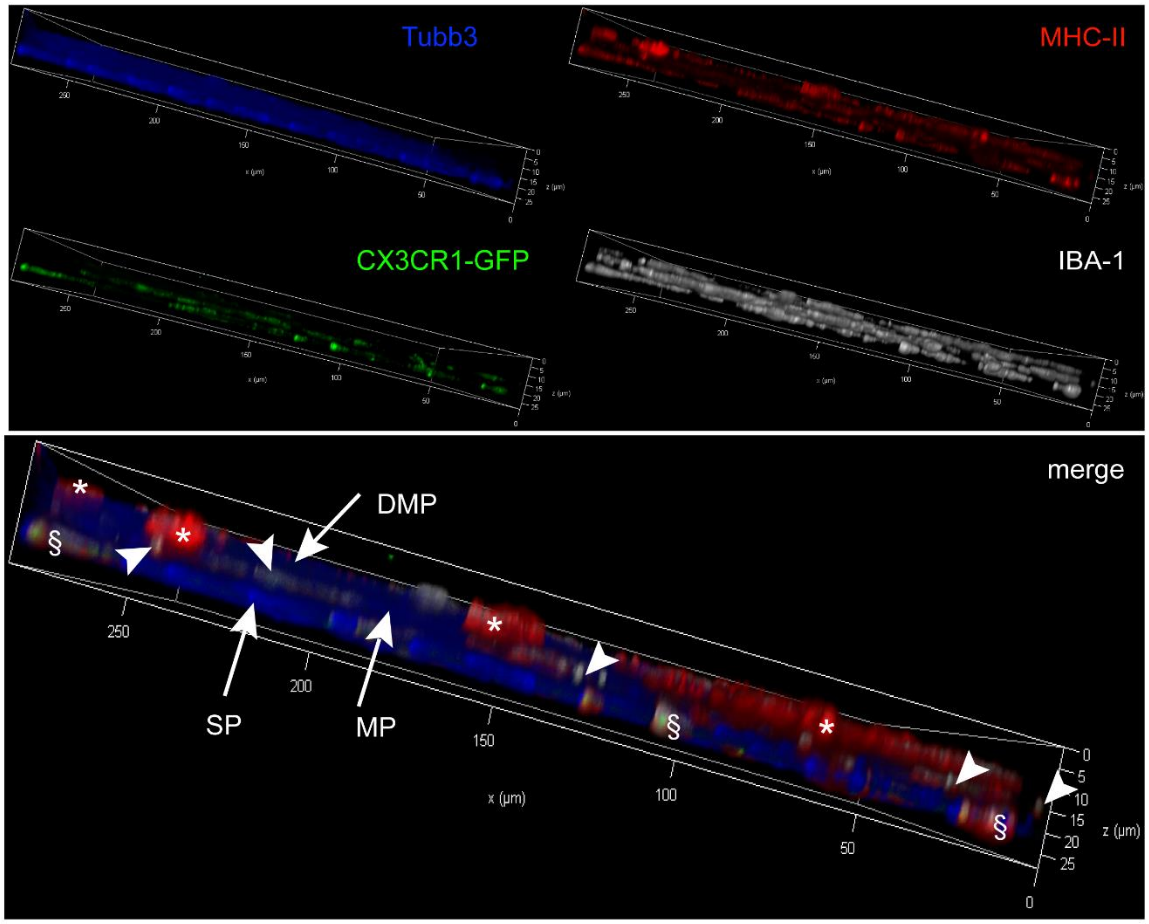

Supplementary Figure S7. Representative 3D projection of stainings for βIII-tubulin (blue), MHCII (red), GFP (green) and IBA-1 (grey) demonstrating the prominent network of MHCII^{+}CX3CR1^{+} and MHCII^{+}CX3CR1^{-} cells located in small intestine whole mounts of the muscularis externa in CX3CR1$^{GFP/+}$. Note that MHCII^{+}CX3CR1^{-} cells (*) lay in a different layer, the deep myenteric plexus (DMP), while MHCII^{+}CX3CR1^{+} cells are located in the myenteric (MP, arrow heads) and serosal (SP,§) plexus. Presence of three individual layers can also be nicely observed in the individual IBA-1 and MHCII stainings while the CX3CR1-GFP staining only shows two cell layers. Notably, all MHCII^{+} cells are also IBA-1^{+}, indicating that these cells are resident macrophages. (Representative image taken from two individual experiments).

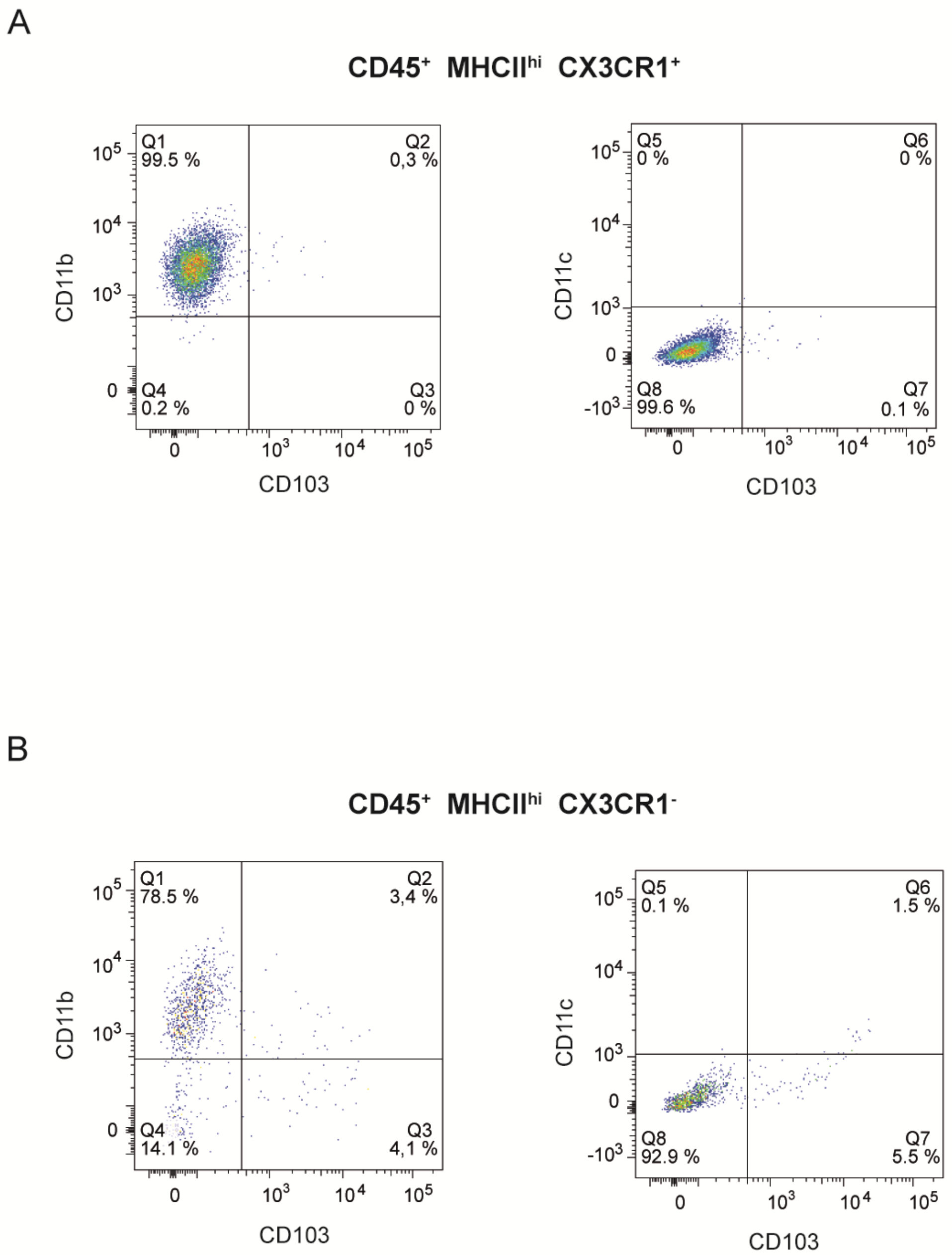

Supplementary Figure S8. Representative FACS staining of the expression of CD11b, CD103 and CD11c on (**A**) Near-IR$^-$CD45$^+$MHCII^{hi}CX3CR1$^+$ and (**B**) Near-IR$^-$CD45$^+$MHCII^{hi}CX3CR1$^-$ gated ME cells of untreated CX3CR1$^{GFP/+}$ mice. n = 3 (the ME of 3 mice was pooled for each replicate).

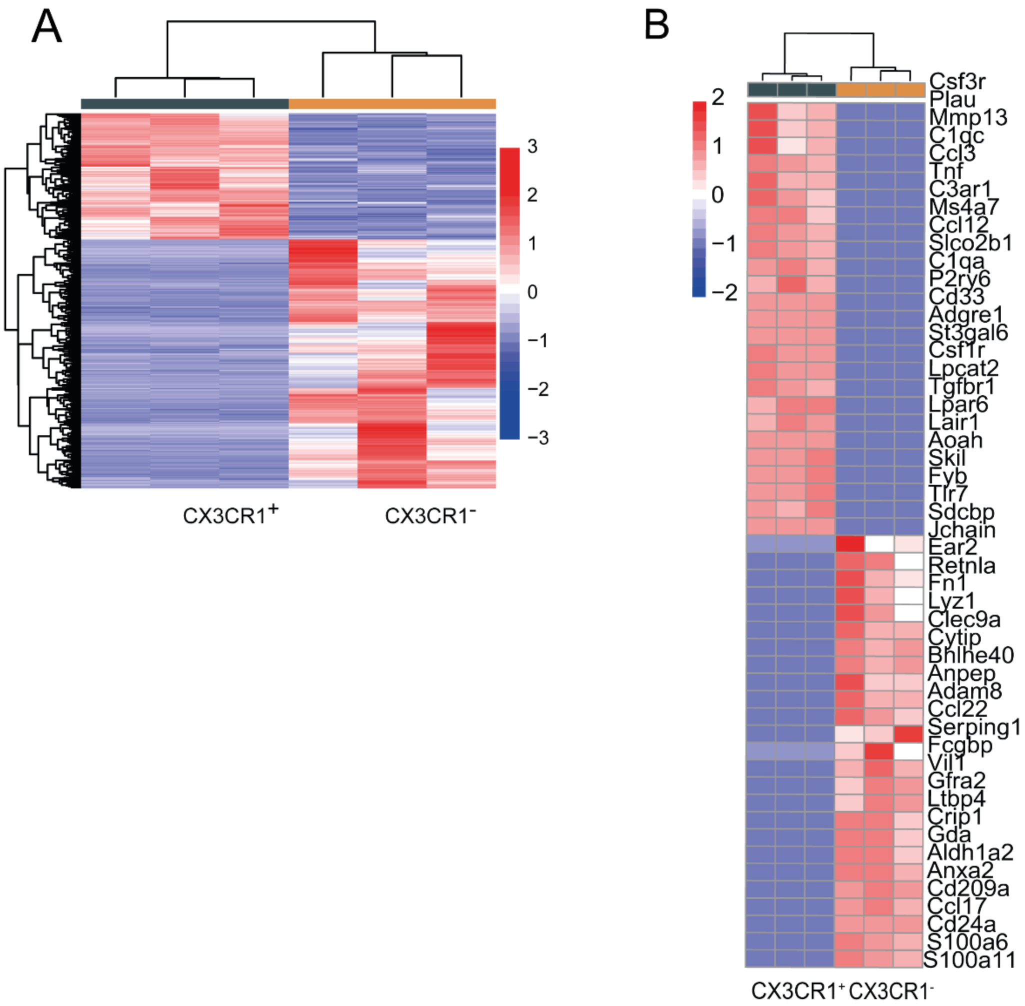

Supplementary Figure S9. MHCII^{hi}CX3CR1^{+} and MHCII^{hi}CX3CR1^{-} cell populations of the ME of naive CX3CR1$^{GFP/+}$ mice were flow cytometry sorted and underwent RNA sequencing. **(A)** Heatmap of the most differentially expressed genes in MHCII^{hi}CX3CR1^{+} and MHCII^{hi}CX3CR1^{-} cells (according to the volcano plot in Figure 6b). (**B**) Heatmap of the top 25 signature genes differentially expressed between MHCII^{hi}CX3CR1^{+} and MHCII^{hi}CX3CR1^{-} cells.
